# Reducing intrusive memories after trauma via an imagery-competing task intervention in COVID-19 intensive care staff: a randomised controlled trial

**DOI:** 10.1038/s41398-023-02578-0

**Published:** 2023-09-01

**Authors:** Lalitha Iyadurai, Julie Highfield, Marie Kanstrup, Alfred Markham, Varsha Ramineni, Boliang Guo, Thomas Jaki, Jonathan Kingslake, Guy M. Goodwin, Charlotte Summers, Michael B. Bonsall, Emily A. Holmes

**Affiliations:** 1 P1vital Products Ltd, Wallingford, Oxfordshire UK; 2 Intensive Care Society, 7-9- Breams Buildings, London, UK; 3 Department of Psychology, Uppsala University, Uppsala, Uppsala County Sweden; 4 NIHR ARC East Midlands, University of Nottingham, Nottingham, UK; 5 MRC Biostatistics Unit, University of Cambridge, Cambridge, Cambridgeshire UK; 6 University of Regensburg, Regensburg, Bavaria Germany; 7 Department of Psychiatry, University of Oxford, Oxford, Oxfordshire UK; 8 Heart and Lung Research Institute, University of Cambridge, Cambridge, Cambridgeshire UK; 9 Department of Biology, University of Oxford, Oxford, Oxfordshire UK

**Keywords:** Psychology, Neuroscience, Psychiatric disorders

## Abstract

Intrusive memories (IMs) after traumatic events can be distressing and disrupt mental health and functioning. We evaluated the impact of a brief remotely-delivered digital imagery-competing task intervention on the number of IMs for intensive care unit (ICU) staff who faced repeated trauma exposure during the COVID-19 pandemic using a two-arm, parallel-group, single-blind randomised controlled trial, with the comparator arm receiving delayed access to active treatment (crossover). Eligible participants worked clinically in a UK NHS ICU during the pandemic and had at least 3 IMs of work-related traumatic events in the week before recruitment. Participants were randomly assigned (1:1) to immediate (weeks 1–4) or delayed (weeks 5–8) intervention access. Sequential Bayesian analyses to optimise the intervention and increase trial efficiency are reported elsewhere [1]. The primary endpoint for the pre-specified frequentist analysis of the final study population compared the number of IMs experienced in week 4 between the immediate and delayed access arms. Secondary outcomes included clinical symptoms, work functioning and wellbeing. Safety was assessed throughout the trial by scheduled questions and free report. All analyses were undertaken on an intention-to-treat basis (86 randomised participants). There were significantly fewer intrusive memories during week 4 in the immediate (median = 1, IQR = 0–3, *n* = 43), compared to the comparator delayed arm (median = 10, IQR = 6–17, *n* = 43), IRR 0.31, 95% CI: 0.20–0.48, *p* < 0.001. After crossover, the delayed arm also showed a significant reduction in IMs at week 8 compared to week 4. There were convergent findings for symptoms of PTSD, insomnia and anxiety, work engagement and burnout, general functioning and quality of life. The intervention was found safe and acceptable to participants. All adverse events were unrelated to the study. Our study provides the first evidence of a benefit on reducing IMs, improving other clinical symptoms, work functioning and wellbeing, as well as safety of a brief remotely-delivered digital imagery-competing task intervention. An efficacy trial with an active control and longer follow-up is warranted. The trial is registered at ClinicalTrials.gov (NCT04992390).

## Introduction

Healthcare staff commonly experience and witness difficult events such as sudden or tragic deaths as part of their daily work which are potentially psychologically traumatic [2]. The frequency of these events has been increased during the COVID-19 pandemic for some clinical areas. A common symptom following a psychologically traumatic event is an intrusive memory related to the event. Such memories spring to mind unbidden—i.e., are involuntarily recalled, replaying of events in the form of sensory-perceptual images - for example, a sudden vivid mental image of a relative’s face at the bedside of a dying patient. Recurrent, involuntary, and distressing intrusive memories of a traumatic event are a core clinical symptom of both acute stress and post-traumatic stress disorder, and a common sub-clinical symptom of these medical conditions [3]. Regardless of whether or not clinical diagnostic criteria for one of these conditions are met, in and of themselves intrusive memories can cause distress, impair concentration and disrupt daily functioning [4].

Intensive care unit (ICU) staff represent a discrete group for whom the adverse impact of the pandemic has been well established. In the UK intensive care units expanded beyond capacity, workforce to patient ratios had to be reduced, staff were unable to deliver the same level of attention to care, and in the first wave there were higher mortality rates and, therefore higher rates of witnessing death, and a lack of family visiting the ICU. Midway through the first wave of the pandemic in 2020, approximately two-fifths of staff working in ICUs in England reported symptoms consistent with a probable mental health disorder including post-traumatic stress disorder (PTSD), anxiety and depression [5]. This represented an increase from pre-pandemic levels in 2017 of approximately 13% [6]. In the first two months of 2021, this figure showed a further increase in the proportion of probable mental disorders in ICU staff, with just under 70% reporting moderate or severe functional impairment [7]. In addition to the current impact on ICU staff, this level of distress also suggests workforce retention could be badly affected in the long term and impact patient care in ICU [7].

To ameliorate the widespread negative impact of the pandemic on the mental health of frontline healthcare staff, effective interventions are urgently needed that can be disseminated quickly and at scale [8]. In addition, clinical working in the NHS continues to be a challenge, and easy to access effective interventions should continue to be available post pandemic. Trials to date have shown benefits of a range of interventions for healthcare workers during the pandemic, including a smartphone app to improve general mental health and wellbeing [9] and guided web-based interventions targeting stress recovery [10] and burnout [11]. There is still a need for interventions targeting symptoms after psychological trauma in the line of work, particularly interventions with longevity and sustainability [12], given the reality of continued exposure to potentially psychologically traumatic clinical events faced by ICU and other frontline healthcare staff.

Existing evidence-based interventions after psychological trauma are clearly highly important [13], though can be challenging to scale up at speed when they include several sessions and/or work with professional therapists/counsellors. Approaches for those with ongoing trauma are limited [13, 14]. To help address the impact after trauma in healthcare staff populations there remains a wider need to additionally develop complementary interventions tools. Approaches that are briefer and simpler may aid adoption in populations working long hours such as healthcare workers during the pandemic. Their work necessitates repeated exposure to traumatic clinical events, as well as time restrictions around shift work, and variation in acceptability of help-seeking for mental health support as that still carries a degree of stigma. More generally, overcoming barriers to provide better support for people after exposure to psychological trauma is a major challenge for treating traumatic stress [15].

We proposed a brief, mechanistically-informed behavioural intervention approach [16] focused on the core symptom of intrusive memories of traumatic events [4, 17, 18], rather than targeting a complex disorder requiring a DSM-5 diagnosis, such as PTSD. The intervention uses a competing cognitive task to disrupt visual mental imagery of traumatic events [19]. It involves a brief trauma reminder cue, followed by playing the computer game Tetris using mental rotation instructions. The intervention overcomes some of the aforementioned challenges to adoption for healthcare staff since: (a) it can be used for single but critically also repeated or ongoing trauma; (b) it is brief (taking about 25 min for each self-guided session), requires only one guided session at the outset, and is low-burden due to the overall low number of sessions required (e.g., one per different intrusive memory); (c) it can be used flexibly at different times and in different locations (e.g., on a smartphone during a commute); and (d) it can be acceptable/non-stigmatising given that it involves a digital task including a computer game rather than talking to a therapist.

We have tested the intervention previously using an experimental medicine approach in a series of controlled laboratory experiments of analogue trauma with non-clinical volunteers, and showed a reduction in the number of intrusive memories in the first week post-intervention after watching a trauma film [20–22]. The approach has been translated to clinical trials with hospital patients soon after a trauma, where the intervention led to a reduction in the number of intrusive memories in the first week after the trauma in the intervention group compared to attention-placebo control [23], and usual care [24]. A further trial showed that the effect extended to one month after trauma [25]. In addition to testing this intervention as a preventative approach soon after trauma (delivered within the first day or so of the trauma), it has also been tested as a treatment approach for people with established intrusive memories of older traumas (when delivered from a few days to many years after trauma), using case series approaches. These studies have shown a reduction in the number of intrusive memories over a 1-week period from pre- to post-intervention in in-patients with complex PTSD [26], refugees [27], women with childhood trauma [28, 29] and in an individual with bipolar disorder [30]. A pilot study with healthcare staff with work-related trauma during the pandemic indicated the approach was feasible and acceptable [31].

This clinical trial advanced on previous trials by testing (a) treatment of established, existing intrusive memories i.e., delivered after a few days to many years post trauma (rather than only as prevention with delivery in the early aftermath of trauma cf. Iyadurai et al., 2018 [23]), (b) an integrated digital version of the intervention, (c) effect on other clinical symptoms and work functioning, (d) use for those exposed to repeated, ongoing trauma (rather than only past trauma) with possible repeat administrations during the study period, and (e) an adaptive optimisation approach [1]. The trial involved a collaborative partnership with the Intensive Care Society (ICS) to help tailor the intervention approach to ICU staff. This collaboration included design of the study, creation of intervention and study materials, user testing of the intervention by ICU staff, recruitment materials and approach via ICS networks, weekly progress meetings, and publication.

We developed a digital version of the imagery-competing task intervention, to facilitate remote delivery under pandemic conditions and for future scalability. All components were integrated on a secure web platform accessed via smartphone, tablet or computer. The intervention package included step-by-step written guidance, instructional video animations, embedded ratings and integrated methods for recording and tracking the number of intrusive memories to guide intervention use. The intervention was delivered in a first session guided by trained researchers, with subsequent sessions self-administered but with optional researcher support.

**Fig. 1:**
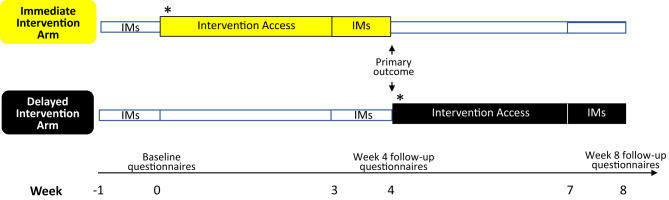
Timeline showing key events in the immediate and delayed arms. Key events for the immediate intervention arm (top timeline in yellow) and delayed intervention arm (bottom timeline in black) are shown across the weekly timepoints (weeks indicated by axis at bottom of figure). Labelled boxes outline when intervention access is received (following baseline week for immediate arm, and following week 4 for delayed arm), and when intrusive memories (IMs) are recorded. Timepoints for questionnaires recorded at baseline, week 4, and week 8, are indicated along the week axis. *The primary outcome* was the between-group comparison of IMs at week 4 indicated by a double ended arrow on the figure. *Secondary outcomes* included within-group comparisons of IMs and follow-up questionnaires, and between-group comparisons of the week 4 follow-up questionnaires.

This paper is complementary to our sequential Bayesian analysis aimed at optimising the imagery-competing intervention and increasing trial efficiency [1]. Here, we report a pre-specified frequentist analyses of the primary and secondary outcomes of the intervention in a two-arm RCT of ICU staff who had experienced traumatic events related to their work during the COVID-19 pandemic (Fig. 1). After recording their intrusive memories during the week before randomisation (baseline), participants in the first arm had *immediate access* to the intervention (one guided session, thereafter self-administered) with symptom monitoring for four weeks (weeks 1–4). Those in the comparator arm had usual care for the first 4 weeks (defined as receiving any treatment they would otherwise access) followed by *delayed access* to the intervention and symptom monitoring for four weeks (weeks 5–8). We selected this comparator (rather than active control) to provide a comparison with real world practice rather than an alternative intervention that is either not used by healthcare staff or not available, and to test for negative as well as positive effects given the novel aspects of this trial.

We hypothesized a reduction in the number of intrusive memories during week 4 (primary outcome) in the immediate arm (intervention) compared to the delayed arm (comparator), controlled for baseline week. Intrusive memories were recorded by participants in a brief daily online diary for 7 days at baseline, week 4, and week 8 (delayed arm). The secondary outcomes included the number of intrusive memories in the delayed access arm at week 8 (within subjects), as well as clinical symptoms, work functioning and wellbeing at weeks 4 and 8 in both arms (between subjects). Adverse events were surveyed throughout the trial by scheduled questions and free report. We also report the acceptability ratings of the participants to the intervention.

## Materials and methods

### Study design

This was a two-arm, parallel-group, single-blind, randomised controlled trial with a comparator arm receiving delayed access to active treatment (crossover). The analyses presented here followed an optimisation phase that used Bayesian adaptive design and sequential analyses, which are presented elsewhere [1]. The Wales Research Ethics Committee 6 approved the study (21/WA/0173), and the trial was prospectively registered with clinicaltrials.gov (CTR: NCT04992390). The protocol was added to the Open Science Framework (OSF) public depository (osf.io/2xn5m) and a Data Monitoring Committee (DMC) established.

Inclusion criteria: Adults aged ≥18 years or older; worked in a clinical role in a National Health Service (NHS) ICU or equivalent during the COVID-19 pandemic; experienced at least 1 work-related traumatic event meeting DSM-5 criterion A for PTSD (“exposure to actual or threatened death, serious injury, or sexual violence” by “directly experiencing the traumatic event(s)” or “witnessing, in person, the event(s) as it occurred to others”); had intrusive memories of the event(s) and experienced ≥3 intrusive memories in the week before screening; had internet access; willing and able to be contacted by the research team during the study; willing and able to provide informed consent and attempt study procedures; and could speak, read and write English. Exclusion criterion was having fewer than three intrusive memories in the baseline week. Receipt of treatments for PTSD symptoms was not an exclusion criterion.

Participants were recruited via email and social media advertisements sent to the ICS membership network as described [1]. Advertisements provided a URL (https://www.p1vital-gains.com/) which included a summary, explanatory video and participant information. Those who were interested completed a brief online screening questionnaire with a check-box for consent. Those meeting inclusion criteria were asked to provide contact details. A researcher contacted them to meet by video call, during which eligibility criteria were verified and written informed consent was obtained from all participants via electronic signature.

### Randomisation

The two arms of the study were: (1) intervention arm: immediate access to the intervention with symptom monitoring for 4 weeks; and (2) comparator arm: delayed intervention i.e., usual care for 4 weeks (defined as receiving any treatments they would otherwise access) followed by access to the intervention/symptom monitoring for 4 weeks. Participants were randomised using a remote, secure web-based clinical research system (P1vital® ePRO) and assigned to either the immediate intervention or comparator (delayed intervention) arm. Initial allocation was set to 66% and amended to 85% after 61 participants had been randomised to achieve balance [1].

Participants and the trial statistician (BG) were blinded to group allocation. Outcome assessments were digitally self-reported by participants (remotely and independently of a researcher) directly into the P1vital^®^ ePRO system (a secure web-based clinical research system which schedules and collects assessment data from participants from either a smartphone, tablet or computer). The P1vital^®^ ePRO system also automatically scored the data. Researchers facilitating the guided intervention sessions were not blinded. No assessments were scored by the unblinded researchers involved in the intervention.

### Intervention

The guided intervention session was delivered remotely by a team of two clinical psychologists and a research assistant. All had received an online training course including theoretical background, key components of the intervention and procedures for effective delivery. Training with experienced clinical researchers (MK and EH) on the intervention and the intrusive memory diary included role-play practice, discussion, and feedback on videoed sessions until staff met competency criteria. Initial participant sessions were observed in real-time by LI. A sample of later sessions were recorded to aid protocol adherence. Staff had weekly group supervision and real-time support as needed via telephone/video call with MK and EH.

After randomisation the researcher contacted participants in the immediate intervention arm to arrange a first guided session with the digital intervention platform, and this was carried out after 4 weeks for the comparator arm (delayed intervention). The intervention consisted of key components integrated on a secure web-based mental health and wellbeing platform (i-spero^®^), to allow remote delivery via smartphone or computer (both in the first guided session by the researcher and subsequently as needed by the participant). The researcher-guided session lasted approximately 1 h, was conducted with the researcher present via Microsoft Teams, and consisted of step-by-step instructions, explanatory videos and multiple-choice questions. Participants were supported to list briefly their intrusive memories in the first session only by typing a short description (not in detail). Then they chose one memory from their list and were asked to bring the image briefly to mind. After receiving instructions, they played the Tetris^®^ game intervention using mental rotation, for 20 min. Thus, the intervention took approximately 25 min each time and they could target different memories on different days. Finally, they were trained in monitoring intrusive memories in daily life using i-spero^®^. For the next 4-weeks they had independent access to the intervention, with the option for researcher support when needed. As part of the Bayesian adaptive design, the intervention underwent an optimisation round to enhance usability of the digital interface [1].

### Assessments

#### Baseline intrusive memory diary

At baseline, participants completed a daily online intrusive memory diary for 7 days to record the number of intrusive memories to work-related traumatic events. This diary was adapted from previous studies [22, 23] for digital delivery using P1vital^®^ ePRO. Intrusive memories were defined as “mental images from a traumatic event that pop suddenly into your mind when you don’t want them to”. Participants watched an animated instruction video which explained “These often take the form of a visual image like a picture in your mind’s eye. They can be vivid or very short and fleeting or broken up or they can be like a movie scene playing. They usually happen when you are awake in the day, or night.” It also explained “intrusive memories are not the same as deliberately choosing to think about the event. They are also not the same as going over and over the event in your mind like thinking in words ‘why did this happen to me?’” Presence of intrusive memories according to this definition was checked by a researcher both during enrolment and the first guided session.

In the intrusive memory diary, participants recorded the number of intrusive memories they had each day, using the following questions: “Have you had any intrusive memories on [date]?” (yes/no response) and if yes, “Please select how many intrusive memories you have had” (− 0 +; participant clicks −/+ to select a number). Reminders to record were sent daily by email. Researchers were available to support participants with any queries or uncertainties about the definition of intrusive memories. Note that in this trial we restricted the daily measure to a simple symptom count to reduce burden to participants (i.e., we did not add further cognitive judgement tasks such as memory distress or vividness ratings each time they made an entry in their diaries and aimed by so doing to enhance the completion rate of the primary outcome measure).

Those who reported at least 3 intrusive memories during the baseline week were asked to complete questionnaires (see below) and were randomised. The total number of intrusive memories that each participant recorded was included as a baseline covariate in the statistical modelling.

#### Baseline questionnaires

Questionnaires completed after the baseline week intrusive memory diary included demographics, work and employment details, health/illness background, experiences of trauma during the pandemic (number of work-related and non-work-related traumatic events, types of work-related traumatic events, timeframes, perceived life threat to someone else/self, peritraumatic distress measured using the Peritraumatic Distress Inventory [32]), and expectancy of treatment effect using the Credibility and Expectancy Questionnaire [33]. Questions used to assess each item are shown in Table 1.

**Table 1:** Summary table of baseline characteristics for ITT population (*n* = 86).

	Delayed arm (*n* = 43)		Immediate arm (*n* = 43)		Both arms combined (*n* = 86)	
DEMOGRAPHICS						
Age and gender	Mean	s.d	Mean	s.d	Mean	s.d
Age (years)	39.9	9.9	37.4	9.8	38.7	9.9
Gender	*n*	%	*n*	%	*n*	%
Woman	35	83.3%	34	81.0%	69	82.1%
Man	7	16.7%	8	19.1%	15	17.9%
Other categories^a^	0	0	0	0	0	0
Highest level of education						
Secondary school (to age 16)	1	2.4%	0		1	1.2%
Sixth form or equivalent (to age 18)	1	2.4%	3	7·1%	4	4.8%
Bachelor’s degree or equivalent	23	54.8%	24	57·1%	47	56.0%
Master’s degree	13	31.0%	11	26·2%	24	28.6%
Doctoral degree	3	7.1%	3	7·1%	6	7.1%
Prefer not to answer	1	2.4%	1	2·4%	2	2.4%
Ethnicity						
White – British	18	42.9%	18	42.9%	36	42.9%
White – Irish	1	2.4%	1	2.4%	2	2.4%
White - Any other White background	14	33·3%	9	21.4%	23	27.4%
Mixed - Any other mixed background	0		2	4.8%	2	2.4%
Asian – Indian	3	7.1%	2	4.8%	5	6.0%
Asian - Any other Asian background	0		1	2.4%	1	1.2%
Black – African	1	2.4%	1	2.4%	2	2.4%
Other - Any other ethnic group	1	2.4%	0		1	1.2%
Unknown - Not stated	4	9.5%	8	19.1%	12	14.3%
Other categories^b^	0	0	0	0	0	0
Marital status						
Single	13	31.0%	13	31.0%	26	31.0%
Living apart from partner	0		1	2.4%	1	1.2%
Married or cohabiting	28	66.7%	25	59.5%	53	63.1%
Divorced or separated	1	2.4%	3	7.1%	4	4.8%
Other categories^c^	0	0	0	0	0	0
Work and Employment						
Work time (hours per week)	37.8	9.2	37·9	13.3	37.8	11.4
Time as healthcare professional (years)	16.4	10.8	13·0	8.6	14.7	9.8
Employment status	*n*	%	*n*	%	*n*	%
Working full time	32	76.2%	34	81.0%	66	78.6%
Working part time	10	23.8%	6	14.3%	16	19.1%
Sick leave	0		1	2.4%	1	1.2%
Other	0		1	2.4%	1	1.2%
Other categories^d^	0	0	0	0	0	0
NHS Job Role^e^						
Allied Health Professionals	1	2.3%	3	7.0%	4	4.7%
Doctors	11	25.6%	8	18.6%	19	22.1%
Health Informatics	1	2.3%	0		1	1.16%
Healthcare Support Worker	1	2.3%	2	4.7%	3	3.5%
Nursing	28	65.1%	29	67.4%	57	66.3%
Other/unknown	1	2.3%	0		1	1.16%
Pharmacy	0		1	2.3%	1	1.2%
Prior Health and Trauma						
Health background^f^	*n*	%	*n*	%	*n*	%
Do you have any current physical health problems e.g., diabetes, heart problems? (yes/no; n = yes)	9	21.4%	8	19.5%	17	20.5%
Have you been treated for/diagnosed with any mental health problems e.g., depression, anxiety, post-traumatic stress disorder? (yes/no; n = yes)	24	57.1%	22	53.7%	46	55.4%
Are you receiving any current treatments/medications for these? (yes/no; n = yes)	17	40.5%	15	36.6%	33	39.8%
Has anyone in your close family been treated for/diagnosed with any mental health problems? (yes/no; n = yes)	16	38.1%	14	34.2%	30	36.1%
Experiences of Prior Trauma	Mean	s.d.	Mean	s.d.	Mean	s.d.
How many work-related traumatic events have you experienced/witnessed during COVID-19? Remember: A traumatic event is defined as an event that involved actual or risk of death, serious injury, or sexual violence for you or someone else.”	38.4	80.9	35.9	58.0	37.1	70.0
How many traumatic events that were not work-related have you experienced/witnessed during COVID-19 (e.g., serious accident, assault, injury or illness)?	10.2	46.2	4.5	15.6	7.3	34.4
Which of the following categories best fit the work-related traumatic events that you have experienced or witnessed during COVID-19, of which you have intrusive memories?	*n*	%	*n*	%	*n*	%
A traumatic or tragic death of a patient	40	95.2%	38	90.5%	78	92.9%
A severe or unsuccessful resuscitation	32	76.2%	26	61.9%	58	69.1%
Witnessing events surrounding colleague who has fallen ill or died of COVID-19	18	42.9%	19	45.2%	37	44.1%
Situation where the care of a patient failed or did not go as planned	31	73.8%	33	78.6%	64	76.2%
Threats or violence against healthcare professionals	13	31.0%	26	61.9%	39	46.4%
Event involving sudden increased risk of COVID-19 infection	29	69.1%	23	54.8%	52	61.9%
A traumatic or tragic event where a patient reminded you of yourself, a family member or friend	31	73.8%	29	69.1%	60	71.4%
Event involving extremely distressed/grieving relatives of patients	33	78.6%	37	88.1%	70	83.3%
Being faced with suicide / suicide attempt	18	42.9%	14	33.3%	32	38.1%
Other	2	4.8%	5	11.9%	7	8.3%
Timeframe of work-related traumatic events experienced at baseline						
Within the last 24 h	4	9.5%	3	7.1%	7	8.3%
Within the past month	8	19.1%	7	16.7%	15	17.9%
Between 1–3 months ago	7	16.7%	8	19.1%	15	17.9%
More than 3 months ago	24	57.1%	22	52.4%	46	54.8%
Ongoing exposure to traumatic events is part of my job during the COVID-19 pandemic	37	88.1%	33	78.6%	70	83.3%
	Mean	s.d.	Mean	s.d.	Mean	s.d.
Perceived life threat to someone else^f^	8.3	2.8	9.1	1.4	8.7	2.3
Perceived life threat to self^f^	5.3	3.4	4.7	3.4	5.0	3.4
Peritraumatic Distress Inventory Total Score^f^	28.6	9.9	30.5	10.2	29.6	10.0
Experiences of Ongoing Trauma						
Week 4^g^	*n*	%	*n*	%	*n*	%
Have you experienced or witnessed any new work-related traumatic events? (yes/no, n = yes)	19	50.0%	11	39.3%	30	45.5%
	Mean	s.d.	Mean	s.d.	Mean	s.d.
How many new work-related traumatic events have you experienced/witnessed?	3.2	6.7	1.6	2.8	2.6	5.5
How many new traumatic events that were not work-related have you experienced/witnessed?	0.2	0.6	0.1	0.5	0.2	0.5
Week 8^h^	*n*	%	*n*	%	*n*	%
Have you experienced or witnessed any new work-related traumatic events? (yes/no, n = yes)	9	29.0%	16	51.6%	25	40.3%
	Mean	s.d.	Mean	s.d.	Mean	s.d.
How many new work-related traumatic events have you experienced/witnessed?	1.5	3.8	2.3	3.5	1.9	3.7
How many new traumatic events that were not work-related have you experienced/witnessed?	0.1	0.3	0.4	1.08	0.2	0.8
Expectancy of Intervention Effect^f^						
	Mean	s.d.	Mean	s.d.	Mean	s.d.
Credibility and Expectancy Questionnaire total score	32.6	8.7	31.6	6.8	32.1	7.8
At this point, how logical does the intervention offered to you seem?	6.0	1.9	5.8	1.6	5.9	1.7
At this point, how successful do you think this intervention will be in reducing your intrusive memories?	5.4	1.7	5.0	1.0	5.2	1.4
How confident would you be in recommending this intervention to a friend who experiences similar problems?	5.9	2.0	5.3	1.6	5.6	1.9
By the end of the intervention period (4 weeks), how much improvement in your intrusive memories do you think will occur? (%)	50.7	21.1	52.7	21.3	51.7	21.1
At this point, how much do you really feel that the intervention will help you to reduce your intrusive memories?	5.2	1.6	5.2	1.6	5.2	1.6
By the end of the intervention period (4 weeks), how much improvement in your intrusive memories do you really feel will occur? (%)	49.0	22.0	50.7	22.5	49.9	22.1

In this table, data for how many new work-related traumatic events have you experienced/witnessed and how many new traumatic events that were not work-related have you experienced/witnessed for week 4 and week 8 are given as the mean (sd). In the Ramineni et al. [1] paper, the data are reported as number (%) with scores categorised into ranges which are 0, 1–5, 6–10, 11–15 and 15+;

The data are presented with missingness excluded and are the mean (SD) or number (%), with percentages calculated according to the number of participants for whom data are available out of the intention-to-treat population (*n* = 86). Data are also shown in Ramineni et al. [1] which has missingness included, and has percentages calculated out of the intention-to-treat-population (*n* = 86).

Total *n* observed for all other data is 84 (delayed arm *n* = 42; immediate arm *n* = 42).

*ITT* Intention to treat, *SD* standard deviation, *n* number.

^a^Other categories for gender were Transwoman; Transman; Gender-variant/non-binary; Other Identity; Prefer not to answer.

^b^Other categories for ethnicity were Mixed - White and Black Caribbean; Mixed - White and Black African; Mixed - White and Asian; Asian – Pakistani; Asian – Bangladeshi; Black – Caribbean; Black - Any other Black background; Other – Chinese; Other – Arab; Other – Traveller.

^c^Other categories for marital status were Widowed; Other.

^d^Other categories for employment status were Jobseeking; Student; Retired.

^e^Data where total n observed = 86 (delayed arm *n* = 43; immediate arm *n* = 43).

^f^Data where total n observed = 83 (delayed arm *n* = 42; immediate arm *n* = 41).

^g^Data where total n observed = 66 (delayed arm *n* = 38; immediate arm *n* = 28).

^h^Data where total n observed = 62 (delayed arm *n* = 31; immediate arm *n* = 31).

#### Primary outcome

During week 4 of the study in both the immediate intervention arm and comparator (delayed intervention) arm, participants were asked again to complete the online intrusive memory diary for 7 days (days 22–28 following the guided session in the immediate arm) to record the number of intrusive memories per day (Fig. 1). The primary outcome was a comparison of the total number of intrusive memories recorded by participants in each arm during week 4, controlled for baseline week (days 0 to 6).

#### Secondary outcomes

For participants in the delayed (comparator) arm only, a secondary outcome was the total number of intrusive memories recorded during week 8 (i.e., after crossover from usual care for 4 weeks to intervention access for 4 weeks). Other secondary outcomes were completed by participants in both arms at baseline, 4 weeks and 8 weeks. Symptoms of PTSD, insomnia, depression, anxiety and post-trauma distress were measured respectively by the PTSD Checklist for DSM-5 (4-item version) [34], Sleep Condition Indicator [35], Patient Health Questionnaire-2 [36], Generalised Anxiety Disorder-2 scale [36, 37], and Impact of Events Scale-Revised (intrusion, avoidance, hyperarousal subscale and total scores) [38]. Work functioning was assessed using engagement and burnout subscales of the Scale of Work Engagement and Burnout [39], number of sick days taken in the previous 4 weeks [40], and intention to leave their job [41]. Wellbeing was categorised by quality of life measured by the 5-level EQ-5D [42], general functioning measured using the 12-item World Health Organization Disability Assessment Schedule [43] and impact of participant-identified problems measured using the Psychological Outcome Profiles [44]. Intrusive memory ratings (e.g., distress, disruption to concentration and impact on work functioning) were assessed using a bespoke 9-item questionnaire.

#### Other outcomes

Changes to health and work were assessed at weeks 4 and 8, including occurrence of new work-related and non-work-related traumatic events, as well as additional stressful life events, new treatments received, and changes to the job or working hours. After weeks 4 and 8 in the immediate and delayed arms, respectively, participants completed a feedback questionnaire with acceptability ratings about the intervention (0 = not at all to 10 = very acceptable). Other measures were number of day/night shifts worked (weekly work pattern) and support from managers and from family/friends.

#### Safety

All participants were asked to report adverse events, including any additional stressful life events or regarding new treatments, with both passive (could be reported throughout the trial) and active surveillance (open ended questions at 4 and 8 weeks).

### Statistical analysis

This study used a Bayesian adaptive design to determine the final sample size (up to a maximum of 150 participants), employing a sequential Bayesian design with maximal sample size [45, 46]. Based on positive evidence from the sequential Bayesian analyses reported elsewhere [1] and following DMC recommendations to the trial steering committee (see Supplementary Material), recruitment concluded early, prior to the planned maximal sample size of 150 [1], with a final number of 86 randomised participants (43 per study arm).

Frequentist analyses were completed in Stata (version 17) and used an intention-to-treat (ITT) population, defined here as all randomised participants (*n* = 86). Note, for the sequential Bayesian analyses [1], time series and expectation maximisation [47] methods were used to impute missing intrusive memory values in the daily online diary where there was partial completion, leading to final Bayesian analysis on 75 participants [1] who had full or partial primary outcome data. Here, we did this and additionally used multiple imputation to handle missing data allowing all 86 randomised participants to be included in our analyses (see Supplementary Materials).

Frequentist statistical analyses were conducted for primary and secondary outcomes in accordance with our statistical analysis plan (osf.io/2xn5m; Descriptive Statistical Analysis Plan). Descriptive statistics were presented as frequencies and percentages (out of non-missing) for categorical variables. The mean and standard deviation and/or median and first and third quartiles (interquartile range; IQR) were presented for continuous variables. All statistical tests were two-sided with a 5% significance level. Treatment effect size estimates and corresponding 95% confidence intervals (CIs) were presented for between-group comparisons and within-group changes.

Between-group comparisons were conducted to determine the effect of the intervention plus symptom monitoring for 4 weeks on primary and secondary outcomes at 4 weeks. Additionally, within-group comparisons were conducted to detect differences in number of intrusive memories before and after the intervention. Likewise, within-group comparisons were performed to determine treatment effects of the intervention on other secondary outcomes before and after the intervention in the immediate intervention and delayed access (comparator) arms.

For the primary outcome analysis, a Zero Inflated Negative Binomial (ZINB) regression model was used with the baseline measure and binary arm status included as covariates, along with binary arm status as a covariate for the zero-inflated part of the model. This was chosen following a numerical model comparison (AIC, BIC) and inspection of a visual plot of difference of observed and predicted values (model fit of general Poisson, Zero Inflated Poisson, Negative Binomial, and Zero Inflated Negative Binomial were compared).

Single level general Poisson regression was used to quantify within arm changes of number of intrusive memories pre- to post-intervention. For other secondary outcomes, multilevel modelling (MLM) was performed with baseline measure, binary arm status, follow-up time and arm-by-time interaction included as fixed-effect covariates, and individual participant taken as a level-two analytical unit [48], to quantify: (a) between-group treatment effect estimates for secondary outcome measures; and (b) within-group changes between each follow-up time for secondary outcome measures. Multilevel (ML) linear regression was applied for normally-distributed continuous data [49], while ML logistic regression was used for binary outcomes, and ML Poisson regression was performed for count data. Likelihood-ratio tests were used to compare between one-level regression, as well as negative binomial regression for count data, with the best fit used as final model [50]. No multiplicity adjustments were applied as there was only one primary outcome, and secondary outcomes aimed to support the primary analyses [51].

## Results

### Recruitment, intervention adherence, outcome completion and attrition

**Fig. 2:**
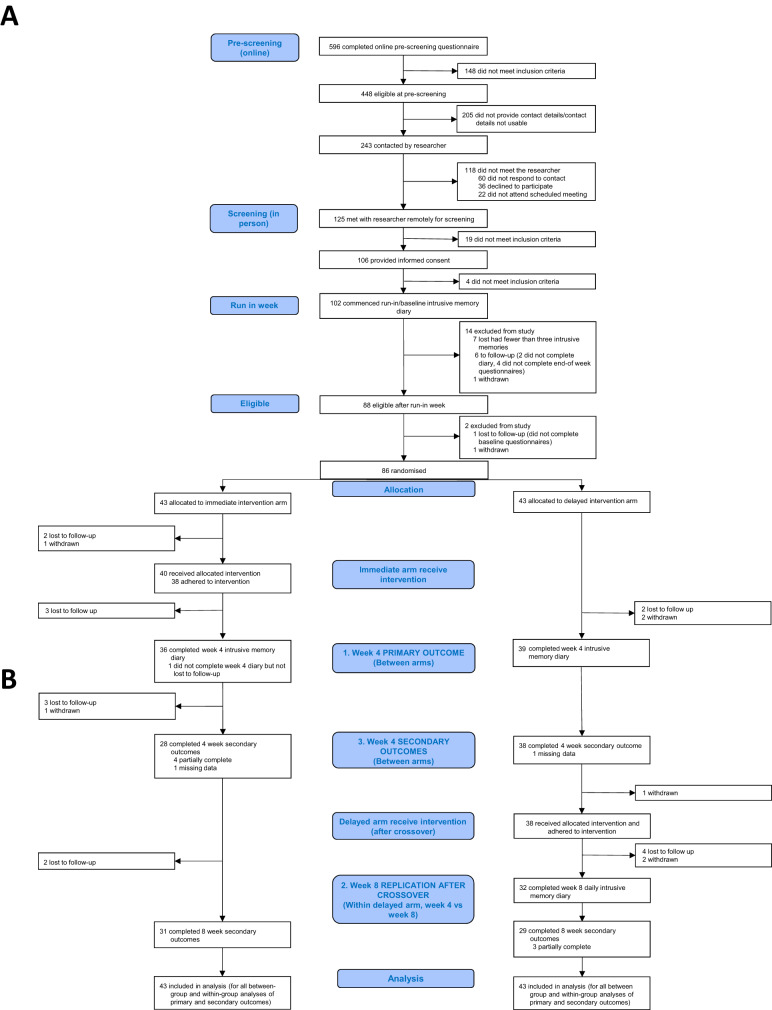
Trial Profile. CONSORT diagram showing enrolment, allocation, primary and secondary outcomes, and analysis populations. **A** Enrollment and Allocation. CONSORT diagram showing enrollment and allocation, as is presented in Ramineni et al. [1]. **B** Primary and Secondary Outcomes. CONSORT diagram showing primary and secondary outcomes at week 4 and week 8. The blue boxes labelled 1–3 outline the main primary and secondary comparisons. 1. Primary analysis: between-group comparison of intrusive memories at week 4. 2. Secondary analyses: within-group comparisons in the delayed arm from week 4 to week 8 (after crossover). 3. Secondary analyses: between-group comparisons of other secondary outcomes at week 4.

A total of 86 participants were randomised between Aug 16, 2021 and Apr 19, 2022 (Fig. 2). Out of these 86 participants (43 per arm, taken as analysis population), 76 met criteria for intervention adherence across both arms (38 per arm, Fig. 2), and 75 had full or partially completed primary outcome data at week 4. Self-reported accuracy ratings of primary outcome completion were high and similar between arms (Supplementary Table 1). Attrition is shown in Fig. 2.

### Baseline characteristics, expectancy and experience of traumatic events

Baseline characteristics were similar between arms (Table 1). Participants had a combined mean age of 38.7 (±9.9) years and were predominantly women, worked full time, and had nursing roles.

At baseline, expectancy of the intervention working was modest (combined mean of both arms = 32.1 (±7.8) on a scale ranging from 6–54. The proportion of the sample reporting current mental health problems was 55.4%.

ICU staff reported high numbers of traumatic events during the pandemic (prior to enrolment in this study) that were both work-related (mean 35.9 (±58.0) and 38.4 (±80.9) in immediate intervention and delayed comparator arms, respectively) and non-work-related (mean 4.5 (±15.6) and 10.2 (±46.2) in immediate intervention and delayed comparator arms, respectively). These were experienced as recently as within the last 24 h (8.3% observed) to over 3 months before (54.8% observed).

**Fig. 3:**
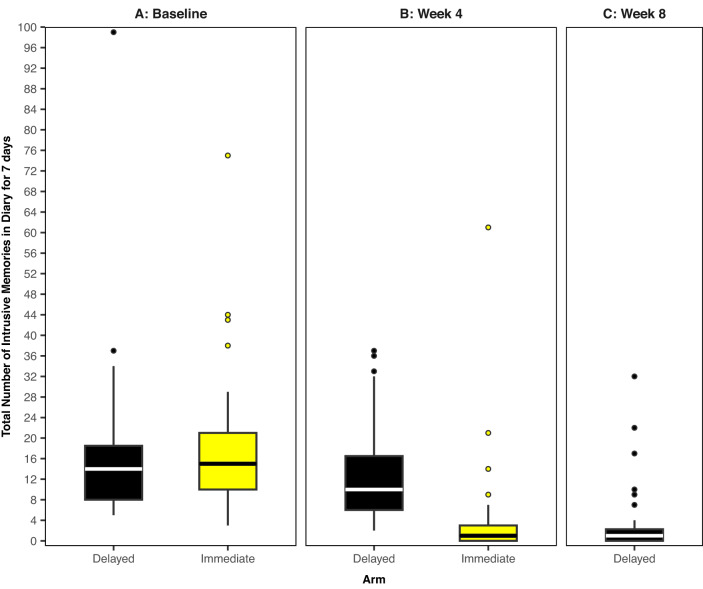
Boxplots showing number of intrusive memories of traumatic events. The midline of each boxplot is the median value, with the upper and lower limits of the box being the third and first quartile (75th and 25th percentile), and the whiskers covering 1.5 times the interquartile range (IQR). The dots depict outliers (each dot represents one participant that departed by more than 1.5 times the IQR above the third quartile and below the first quartile). All outliers are included in this figure. Further to a version of this figure presented in Ramineni et al. [1], we now include the secondary outcome measure (week 8 data for the delayed arm i.e., data after the delayed arm receive access to the intervention for four weeks), as well as inclusion of a participant in the immediate arm who only completed the baseline measure. **A** Baseline measure for each arm. Number of intrusive memories of traumatic events recorded by participants in a brief daily online intrusive memory diary for 7 days during the baseline week for both arms (black = delayed arm; *n* = 39: usual care for four weeks; yellow = immediate arm; *n* = 37: immediate access to the intervention following the baseline week), showing that the two arms did not differ at baseline (i.e., before the intervention was provided to either arm). **B** Primary outcome measure for each arm. Number of intrusive memories of traumatic events recorded by participants in the daily online intrusive memory diary for 7 days during week 4 for each arm (black = delayed arm; *n* = 39: usual care for 4 weeks; yellow = immediate arm; *n* = 36: immediate access to the intervention following the baseline week). The intervention consisted of a cognitive task involving a trauma reminder-cue plus Tetris^®^ computer gameplay using mental rotation plus symptom monitoring. The immediate access arm had fewer intrusive memories at week 4 compared to the delayed arm, and the number of intrusive memories for the immediate arm decreased between the baseline week and week 4. **C** Secondary outcome measure for the delayed intervention arm. Number of intrusive memories of traumatic events recorded by participants in a brief daily online intrusive memory diary for 7 days during week 8 for the delayed arm (black; *n* = 32: usual care for four weeks followed by access to the intervention for 4 weeks), showing that the number of intrusive memories decreased between week 4 and week 8.

The number of intrusive memories in the baseline/run-in week was similar between trial arms (combined median = 14, IQR = 9–20) (Table 2 and Fig. 3).

**Table 2:** ITT analysis results for number of intrusive memories (treatment effects for between-arm comparison of primary outcome, and within delayed arm comparison of secondary outcome).

Number of Intrusive Memories (IMs)	Delayed arm (*n* = 39)		Immediate arm (*n* = 36)		ITT Treatment Effect Estimate	
					IRR	95% CI
Primary Outcome (Week 4) – Between Arm Comparison						
Median, IQR	10.00	(6.0, 17.0)	1.00	(0.0, 3.0)	0.31***	0.20, 0.48
Mean, SD	12.46	9.28	4.03	10.68		
Secondary Outcome (Week 8, after crossover^a^) - Within Delayed Arm Comparison						
Median, IQR	1.00	(0.0, 2.50)			0.31***	0.21, 0.45
Mean, SD	3.78	7.23				
Baseline/Run-In Week						
Median, IQR	14.00	(8.0, 19.0)	14.50	(10.0, 21.50)		
Mean, SD	17.08	15.84	18.50	13.61		

Treatment effects presented for between-arm comparison of number of intrusive memories at week 4 (primary outcome), and within delayed arm comparison of number of intrusive memories at week 8 (after crossover) from week 4. Median (IQR) and Mean (s.d.) of number of intrusive memories at baseline, week 4, and week 8 are also presented.

*ITT* intention to treat, *IRR* incidence rate ratio, *CI* confidence interval, *IQR* interquartile range, *SD* standard deviation.

***p < 0.001;

^a^ITT treatment effect estimate within the delayed arm after crossover (weeks 4–8).

### Primary outcome

Participants in the immediate intervention arm reported significantly fewer intrusive memories during week 4 (median = 1, IQR = 0–3) than those in the comparator (delayed intervention) arm (median = 10, IQR = 6–17) (IRR = 0.31, 95% CI: 0.20–0.48, *p* < 0.001; between-groups analysis; Table 2 and Fig. 3). Sensitivity analyses evaluating the robustness of treatment effect showed this difference remained significant using observed data only, observation-level random effects, exclusion of outliers, and using a pre-specified per-protocol population (Supplementary Fig. 1).

### Secondary outcomes

Within the comparator arm, where participants had delayed access to the intervention (i.e., delayed arm crossover), there was a significant reduction in the number of intrusive memories in week 8 compared to week 4 (within-group analyses, Table 2 and Fig. 3).

Note, within the comparator arm 38 of 39 participants took up the delayed offer of the intervention (Fig. 2). All 38 participants in the comparator arm adhered to the guided intervention session, whilst 38/40 did so in the immediate intervention arm, Fig. 2.

### Other secondary outcomes (between arms)

#### Clinical symptoms

At week 4, the immediate intervention arm participants had significantly lower symptoms of PTSD, insomnia, anxiety and post-trauma distress, with no difference in depression, than the comparator delayed arm (Table 3).

**Table 3:** ITT analysis results for secondary outcomes (treatment effects for between-arm comparisons).

Measure	Delayed arm			Immediate arm			ITT treatment effect estimate (between-arms)	
	*N*	Mean	SD	*N*	Mean	SD	Mean Difference	95% CI
Clinical Symptoms								
PTSD (PCL-5 4-item)								
Baseline	43	8.00	3.27	42	8.71	3.64		
Week 4	38	6.74	2.62	31	4.26	2.86	−2.29***	(−3.52, −1.07)
Insomnia (SCI-08)^a^								
Baseline	42	12.71	6.62	41	12.37	7.75		
Week 4	38	12.71	6.06	30	19.13	8.27	5.38***	(2.56, 8.20)
Anxiety (GAD-2)								
Baseline	42	3.07	1.81	41	3.83	1.80		
Week 4	38	2.84	1.79	30	2.07	1.91	−0.93*	(−1.68, −0.17)
Depression (PHQ-2)								
Baseline	42	2.26	1.82	41	2.76	1.48		
Week 4	38	1.92	1.62	30	1.53	1.50	−0.52	(−1.23, 0.19)
Post-trauma distress (IES-R total)								
Baseline	43	2.17	0.71	41	2.24	0.77		
Week 4	38	1.70	0.56	32	0.83	0.58	−0.8***	(−1.08, −0.53)
Post-trauma distress (IES-R intrusion subscale)								
Baseline	43	2.43	0.80	41	2.52	0.91		
Week 4	38	1.86	0.67	32	0.87	0.63	−0.95***	(−1.27, −0.62)
Post-trauma distress (IES-R avoidance subscale)								
Baseline	43	2.22	0.83	41	2.19	0.81		
Week 4	38	1.95	0.70	32	1.06	0.75	−0.75***	(−1.09, −0.42)
Post-trauma distress (IES-R hyperarousal subscale)								
Baseline	43	1.83	0.93	41	2.03	0.96		
Week 4	38	1.27	0.68	32	0.53	0.52	−0.7***	(−0.97, −0.44)
Work Functioning								
Work Engagement (SWEBO)^a^								
Baseline	42	2.19	0.53	40	2.03	0.46		
Week 4	38	2.10	0.48	28	2.59	0.66	0.46***	(0.20, 0.71)
Work Burnout (SWEBO)								
Baseline	42	2.33	0.50	40	2.45	0.54		
Week 4	38	2.40	0.61	28	1.80	0.54	−0.61***	(−0.87, −0.34)
Sickness Absence (number of sick days over past four weeks)								
Baseline	42	3.21	6.62	40	3.80	6.91		
Week 4	38	3.45	7.61	28	3.79	7.36	1.50^b^	(0.64, 3.51)
Intention to leave job								
Baseline	42	9.19	3.62	40	8.65	3.79		
Week 4	38	10.32	3.35	28	9.36	4.63	−0.86	(−2.20, 0.49)
Wellbeing								
Quality of Life (EQ-5D-5L Scale Score)^a^								
Baseline	42	67.83	19.30	40	66.03	19.40		
Week 4	38	65.13	21.51	28	78.68	13.52	12.32**	(4.61, 20.04)
General functioning (WHODAS 2.0)								
Baseline	42	21.88	14.96	40	25.00	15.23		
Week 4	38	22.37	14.40	28	13.54	11.94	−9.78***	(−15.06, −4.50)
Impact of participant-identified problems (PSYCHLOPS)								
Baseline	42	13.29	2.94	40	14.20	3.30		
Week 4	38	12.13	3.57	30	6.97	4.78	−6.49***	(−8.48, −4.51)
Intrusive Memory Ratings								
How distressing were your intrusive memories?^c^								
Baseline	43	6.00	1.83	42	6.02	1.83		
Week 4	38	5.53	1.94	35	2.86	2.45	−2.63***	(−3.72, −1.54)
How much did they disrupt your concentration?^c^								
Baseline	43	6.70	1.88	42	7.05	2.29		
Week 4	38	5.95	1.72	35	2.74	2.76	−3.21***	(−4.28, −2.15)
How much did they interfere with what you were doing?^c^								
Baseline	43	5.42	2.30	42	5.95	2.26		
Week 4	38	5.13	1.99	35	2.26	2.32	−2.92***	(−3.90, −1.95)
How much did your intrusive memories affect your work functioning?^c^								
Baseline	43	4.53	2.75	42	4.71	2.64		
Week 4	38	3.97	2.81	35	1.71	2.63	−2.15***	(−3.22, −1.08)
How much did your intrusive memories affect your functioning in other areas of your life?^c^								
Baseline	43	5.30	2.53	42	5.55	2.21		
Week 4	38	4.42	2.78	35	1.89	2.35	−2.59***	(−3.67, −1.51)

Treatment effects presented for between-arm comparisons of secondary outcomes at week 4 (clinical symptoms, work functioning, wellbeing, and intrusive memory ratings). Mean (SD), and number N at baseline, and week 4 are also presented.

*ITT* Intention to treat, *SD* standard deviation, *PCL-5* PTSD Checklist for DSM-5 (4-item version), *SCI-08* Sleep Condition Indicator (8-item version), *GAD-2* Generalised Anxiety Disorder Assessment (2-item version), *PHQ-2* Patient Health Questionnaire (2-item version), *IES-R* Impact of Event Scale - Revised, *SWEBO* Scale of Work Engagement and Burnout, *ITL* Intention to Leave Job, *WHODAS 2.0* World Health Organization Disability Assessment Schedule 12-item version, *EQ-5D-5L* European Quality of Life Five Dimension Five Level Scale, *PSYCHLOPS* Psychological Outcome Profiles Questionnaire.

*p ≤ 0.05; **p ≤ 0.01; ***p ≤ 0.001;

^a^Higher score on these measures indicates better functioning (i.e., better sleep, greater work engagement and better quality of life).

^b^For sickness absence, the treatment effect estimate is the Incidence Rate Ratio rather than the mean difference.

^c^Indicates an item where responses were made on an 11-point scale where 0 = not at all to 10 = extremely or very much.

#### Work functioning

Work engagement was significantly higher and burnout significantly lower in participants in the immediate intervention arm compared to those in the delayed arm at 4 weeks. There was no significant difference in number of sick days or intention to leave the job (Table 3).

#### Wellbeing

Quality of life and general functioning were significantly improved in the immediate intervention compared to delayed intervention arm at 4 weeks. The impact of participant-identified problems also showed a significantly better outcome (Table 3).

#### Intrusive memory ratings

At 4 weeks, the immediate intervention arm had lower scores than the comparator (delayed intervention) arm on all ratings regarding impact of intrusive memories: distress, disruption to concentration, interference with current task, impact on work functioning, and impact on functioning in other areas of life (Table 3) as well as frequency and duration of interference (Supplementary Table 2).

### Other secondary outcomes (within-arms)

#### Changes from baseline to 4 and 8 weeks (within immediate arm)

In line with the between-arm differences in secondary outcomes, within-arm changes in the immediate intervention arm from baseline to week 4 showed a similar pattern of significant differences in clinical symptoms, work functioning, wellbeing, and intrusive memory ratings (Supplementary Table 3).

Within-arm changes from baseline to week 8 showed that all significant differences in week 4 had been maintained, and there was now also a significant reduction in depression symptoms and number of sick days (Supplementary Table 3).

#### Changes from week 4 to week 8 after crossover (within delayed arm)

After access to the intervention at week 4, the delayed (comparator) arm showed the same patterns of significant differences at week 8 in clinical symptoms, work functioning, wellbeing and intrusive memory ratings (within-group analyses, Supplementary Table 3), replicating the findings found in the immediate intervention access arm.

### Other outcomes

#### Changes to health and work

New work-related traumas were experienced by 45.5% of respondents at 4 weeks and by 40.3% of respondents at 8 weeks (Table 1). Additional stressful life events, new treatments received, changes to the job and number of hours worked per week are shown in Supplementary Table 4.

#### Feedback questionnaire

Intervention acceptability was high and similar between groups (combined mean rating = 8.5 ± 1.88). Overall, the intervention was considered as easy, helpful, and not too distressing, but slightly burdensome (Supplementary Table 5).

### Other measures

Weekly work patterns are reported in Supplementary Table 6. Support from managers, family and friends at baseline is shown in Supplementary Table 7.

### Safety

All adverse and serious adverse events reported by participants during the study were unrelated to the intervention or trial procedures (Supplementary Table 8).

## Discussion

This paper reports the frequentist analyses of both the primary and secondary outcomes in the final study population of a two-arm, parallel-group randomised controlled trial of a remotely-administered digital intervention seeking to reduce the number of intrusive memories of trauma experienced by ICU staff who worked during the COVID-19 pandemic. One arm received immediate access to the intervention, while the comparator arm received delayed access to the active treatment (crossover). Sequential Bayesian analyses were used to optimise the intervention as reported elsewhere [1] and this paper presents the frequentist analyses of both the primary and secondary outcomes in the final study population in addition to the crossover results from participants receiving delayed (rather than immediate) access to the trial intervention.

The primary outcome was impact on the number of intrusive memories. Staff who had immediate access to the imagery-competing task had approximately one-tenth the number of intrusive memories during week 4 compared to staff who received usual care over the 4-week interval. In addition, ICU staff in the immediate intervention arm had on average a 78% reduction in their number of intrusions in week 4 compared to their baseline, with 36% experiencing zero intrusive memories. Importantly, this finding was further replicated by the comparator arm participants who had delayed access to the trial intervention (crossover design) who had on average a 73% reduction in the number of intrusions in week 8 compared to week 4, with 38% experiencing zero intrusive memories. Moreover, the intrusive memories were found to be less distressing [52] in the immediate intervention arm. Demographics of the sample were broadly representative of the NHS workforce [53, 54].

These results indicate a robust effect despite a number of factors. First, staff generally expressed only modest expectations of the intervention having an effect (as measured by credibility/expectancy ratings) and critically this did not differ between groups. Second, participants were informed that they would receive the intervention at some point in the next 4 weeks but not when, and, therefore, were not aware of the delayed crossover design which maintained blinding. Finally, the two arms showed a similar level of ongoing work-related trauma during each phase of the study (>40%). This indicates the possibility that individuals may benefit from the intervention despite ongoing trauma exposure. In addition, the sensitivity analyses indicated robustness of the treatment effect across different statistical scenarios. This included eliminating outlier effects and the use of a per protocol population that excluded those with protocol deviations.

For the secondary outcomes, we found that participants in the intervention arm who had immediate access to the intervention showed significant reductions in clinical symptoms of PTSD, insomnia and anxiety, as well as increased work functioning and wellbeing at week 4, compared to the those in the comparator arm who had delayed access to the trial intervention. Furthermore, these effects were sustained at week 8 within the immediate arm, and significant reductions in symptoms of depression and sickness absence rates were also observed at this time point compared to baseline. Importantly, the comparator (delayed) arm participants showed the same pattern of changes after crossover, demonstrating replication of the beneficial effects of the intervention on PTSD, insomnia and anxiety symptoms, as well as work functioning and wellbeing. All adverse events were unrelated to the study, no other harms were reported, and no withdrawals due to harms occurred, suggesting the intervention approach was safe.

The finding that a reduction in the number of intrusive memories was also accompanied by an improvement in clinical symptoms related to PTSD and anxiety supports the possibility that changing a single symptom – intrusions - may have a causal influence on other symptoms. Network models of PTSD symptoms suggest intrusion symptoms are centrally-linked and may emerge within connected networks over time [55–58]. It is an intriguing possibility for experimental science-driven treatment development that changing just one symptom following trauma may have a downstream effect on others in the network, and raises potential for this intervention for both treatment and prevention. It has been recently argued [18] that a focus on the specific symptom domain of intrusive memories of trauma (rather than the broader breadth of PTSD symptoms) could advance much-needed novel treatment strategies post-trauma. Here our inclusion criterion was 3 intrusive memories per week. We note that for a PTSD diagnosis, the Clinician-Administered PTSD Scale for DSM–5 (CAPS-5) [59] requires at least *two* intrusive memories over the past month. The CAPS-5 maximum score is ‘daily’, and the mild-minimum score is ‘once-or-twice a week’/‘never’. In this context, we here found the number of intrusive memories in the baseline/run-in week was 14 (combined median) and reduced to 1 in the immediate arm. Further research on the number of intrusions in relation to both inclusion and outcome is warranted.

Questionnaire feedback from staff indicated the intervention was acceptable and easy to use, suggesting that the approach might help overcome some of the challenges that ICU and other healthcare staff can face in accessing mental health support. This includes stigma and low willingness to disclose mental health problems [60]. An approach targeting a single symptom that is self-identified as distressing and disruptive, rather than one that requires a diagnosis of a mental health disorder, could offer advantages. The intervention was used in a self-guided manner with optional researcher support after its initial introduction by the researcher. Its low-resource character offers the opportunity for scalability, and further research is warranted.

Our findings that the two arms showed a similar and high level of ongoing work-related trauma during each phase of the study indicates how this intervention may be of potential use in the context of continued traumatic exposure. At baseline, participants reported having experienced a very high number of traumatic events during the pandemic, which may reflect how working on an intensive care ward during the pandemic has been an unprecedented time for healthcare staff. Further work is needed to benchmark work-related trauma exposure during the pandemic and also pre-pandemic [2]. While this is a “treatment” trial of existing intrusive memories, there is the possibility the intervention may also help with “prevention” in the context of ongoing trauma in that people could, with a self-guided tool, swiftly reduce the build-up of intrusions after a new trauma exposure. Research remains to see whether the approach may also help in the prevention of other clinical symptoms or diagnoses such as PTSD.

In addition, our finding of positive effects of the intervention on work functioning (improved engagement and less burnout), general functioning and quality of life may be important for ICU staff who are continuously exposed to traumatic clinical events, while taking critical decisions for patient care. For example, whilst working with a dying patient, a nurse may suddenly have a vivid intrusive memory of the face of a previous dying patient, which disrupts concentration and brings difficult emotions at a critical time. Reducing the number of intrusive memories, or complete removal of this symptom as seen by over a third of participants in this trial, may therefore be of direct benefit to the individual and their work.

The limitations of this trial include a relatively short follow-up (4 weeks for the primary outcome), and no assessment of the number of intrusive memories at 8-week or longer-term follow-up in the immediate arm. There is a need to determine whether there is persistence of the treatment effect beyond the first month, for both primary and secondary outcomes such as work functioning and quality of life. Longer-term follow up (such as at 3 and 6 months) might allow us to better assess change on measures that are unlikely to show change after only one month, such as sickness absence. We used a usual care control in the delayed arm (where participants accessed any treatments they would otherwise receive) given the novel use of this intervention. Having established positive effects, future trials would benefit from an additional active control comparator with an alternative cognitive task delivered using the same digital platform. Under pandemic conditions, for this first optimisation study we did not wish to withhold a treatment that potentially improves intrusive memories (based on previous studies) and hence a delayed intervention group was used rather than a traditional control group. We have tested our digital intervention as a standalone approach, but future studies could investigate combination approaches with other mental health treatments [60, 61]. A strength in this study was the use of short versions of measures (which have high correlations to long versions [34]) to reduce participants’ burden: however, use of the full 20-item version of the PCL-5 may improve treatment sensitivity in future trials.

Investigation of brain mechanisms of the current intervention is beyond the scope of the current RCT and should be the focus of further work [62–64]. The experience of an intrusive memory (which comprises mental imagery) is thought to disrupt our perceptual systems [65], and conversely occupying visuospatial perceptual systems may disrupt imagery-based memory. That is, mental imagery-interfering tasks (such as playing Tetris) may compete for perceptual resources with other imagery that is brought to mind at that time (e.g., a sensory memory), and thereby weaken that memory’s representation. In turn, if sensory aspects of the memory are weaker this may make the memory less readily triggered by perceptual cues and lead to fewer intrusive memories [66].

To apply this idea to established intrusive memories of older trauma, the procedure has been informed by research on memory updating and so-called memory reconsolidation [67, 68]. Such literature suggests that a reminder cue to bring to mind the content of a specific established intrusive memory of an older event (i.e., the memory hotspot [69, 70]) could act to reactivate that part within the memory and render it labile and open to updating [71]. Thus, if the reminder cue is given prior to the visuospatial task with sufficient time allowed for memories to become modifiable, then even established intrusive memories of older events could be updated [22], at least when the competing task is performed for a sufficient duration for memory updating to have completed. Taken together, this could explain the lasting effects of the intervention for established intrusive memories of older trauma (beyond the initial working memory competition whilst completing the task or for very recent trauma). Clearly, further mechanistic work is needed. The intervention’s task-based procedures may also engage homotypical association areas, including those connected to medial prefrontal cortex [72]. It is a unique ability of humans to “relive” visually past events in the “here-and-now,” accompanied by emotional responses that occurred during memory encoding. While reliving is often advantageous, after trauma this capacity engenders memories that may be intrusive and distressing [72] for those affected by the trauma such as healthcare staff.

In conclusion, we found that a brief, digital imagery-competing task intervention reduced the number of intrusive memories, and improved clinical symptoms, work functioning and wellbeing after 4 weeks, for ICU staff exposed to work-related traumatic events experienced during the COVID-19 pandemic. The intervention was acceptable for ICU staff with ongoing trauma exposure. Findings support progression to an efficacy trial comparing the guided intervention with an active control, using a longer follow-up. This remotely-delivered, brief, flexible, low-intensity intervention offers one potential solution to address the impact of work-related trauma on the mental health and functioning of healthcare workers, with potential for future scalability.

## Licensing and quality statement

The brief digital intervention on i-spero^®^ is owned and manufactured by P1vital Products Ltd. Tetris^®^ has been licensed for use within i-spero^®^ from The Tetris Company. P1vital^®^ ePRO, i-spero^®^ and the brief digital intervention have been developed following a formal computerized system validation methodology which complies with Good Clinical Practice, FDA 21CFR Part 11 and ISO13485 Quality Management System.

### Supplementary information

Supplementary Materials

Supplementary Tables

## Data Availability

Anonymised data, dictionary, analytical code, study protocol and frequentist statistical analysis plan are available on the OSF (https://osf.io/j9v2z/).
