# Supplementary material for: Reducing intrusive memories after trauma via an imagery-competing task intervention in COVID-19 intensive care staff: a randomised controlled trial
Source: Transl Psychiatry. 2023 Sep 1;13:290. doi: 10.1038/s41398-023-02578-0 (PMC10474101; doi:10.1038/s41398-023-02578-0)
Supplement: Supplementary file 1 — Supplementary Materials [file 41398_2023_2578_MOESM1_ESM.docx]

**Supplementary Materials**

**“Reducing intrusive memories after trauma via an imagery-competing task intervention in COVID-19 intensive care staff: A randomized controlled trial”**

Lalitha Iyadurai, Julie Highfield, Marie Kanstrup, Alfred Markham, Varsha Ramineni, Boliang Guo, Prof Thomas Jaki, Jonathan Kingslake, Prof Guy Goodwin, Prof Charlotte Summers, Prof Michael B. Bonsall & Prof Emily A. Holmes.

**Contents**

1. Frequentist Statistical Analyses
   1. Software
   2. Sample Size
   3. Model Framework for Primary Outcome
   4. Model Framework for Secondary Outcomes
   5. Missing Data
   6. Sensitivity Analyses
2. Supplementary Tables
3. References for Supplementary Materials
4. **FREQUENTIST STATISTICAL ANALAYSES**
   1. **Software**

All frequentist statistical analysis was undertaken using Stata (version 17), and additionally Blimp (version 3.0) for missing data imputation.

- 1. **Sample Size**

This study used a Bayesian adaptive design to determine the final sample size (up to a

maximum of 150 participants). Prior to final analysis, sequential Bayesian analyses were conducted (when n=20,23,29,37,41,45 participants had completed the primary outcome) to inform early stopping of the trial prior to the planned maximum recruitment (n=150). From the first analysis (at n=20) there was strong evidence against a negative treatment effect (Bayes Factor, BF=59.8) therefore the trial did not need to be stopped/altered on this basis. Supportive evidence for the positive treatment effect (Bayes Factor > 20 in favour of the hypothesis that those on the immediate arm, compared to the delayed arm have fewer intrusive memories at week 4, as opposed to no-effect) was reached well before the originally proposed sample size had been randomised. Following Data Monitoring Committee’s (DMC) recommendation to the trial steering committee, the trial was concluded early as there was sufficient evidence for the effectiveness of the intervention. Therefore, recruitment concluded early, giving a final number of 86 randomised participants for frequentist ITT analysis (43 per study arm).

- 1. **Model Framework for Primary Outcome**

The primary outcome is based on count data. Therefore, to quantify treatment effect estimate and its 95% CI on primary outcome, Poisson regression was planned to be performed with baseline measure and binary arm status included as fixed effect covariates. To account for modelling assumptions, various regression models for count data (Poisson, Zero Inflated Poisson, Negative Binomial, Zero Inflated Negative Binomial) were also compared to find the best fitting model. Visual exploratory data and model evaluation showed that the Zero Inflated Negative Binomial (ZINB) regression model provided the best fit for our data and was used as the model for the intention-to-treat analysis of the primary outcome.

- 1. **Model Framework for Secondary Outcomes**

To quantify treatment effect estimate and within arm change between each measuring time for the number of intrusive memories at week 8 and other secondary outcome measures, MLM was performed with baseline measure, binary arm status, following up time and interaction of arm × time included as fixed effect covariates, and participant as level 2 analytical units [1] . ML linear regression was used for normally distributed continuous data [2], ML logistic regression was used for binary outcomes. Multilevel Poisson regression was performed for count data. Skew continuous measure were transformed for ML linear regression model if needed with reference on data exploratory results (see for example [3–5]). For count data, ZIP model was performed if there were extreme 0 counts or negative binomial model if the data were over dispersed. The treatment effect estimate and its 95% CI together with significance level for between group comparison were derived from MLM, the estimate and its 95% CI were presented for within group change from baseline to each follow-up time. If MLM result showed non-significant level-two variance estimate, or any model convergence issues, conventional single level regression was performed with cluster-robust standard error reported for treatment effects estimate.

- 1. **Missing Data**

**Intrusive Memory Diary Data**

When genuine missing data occurred in the daily intrusive memory diary, we imputed the missing values using time-series methods [6] and an expectation-maximisation (E-M) algorithm [7]. Initial missing values were imputed by taking expectations across a participant’s available diary data. Using Poisson likelihood and correlated errors, we maximised over this ‘full’ data set to provide updated expected values for the missing data. We iterated over these latter steps until convergence in values of missing data (to a pre-determined threshold) was achieved.

Let $y = (y_{1}, y_{2},y_{3},y_{4}, y_{5},y_{6},y_{7})$be a participant's observed diary data. The maximum likelihood approach for dealing with missing values fits an autoregressive model of order 1 (AR(1) model) regression to the observed data through a simulated annealing algorithm. The main algorithm uses an E-M algorithm to impute missing values based on Poisson time series regression [7]. Given a linear time-series model $y_{t}= a_{1}y_{t-1}+ a_{2}$, the mean of a Poisson distribution is $\lambda= exp(a_{1}y_{t-1}+ a_{2}$), with Poisson probability function

$Pr(y_{t}| y_{t-1}; a_{1}, a_{2}) = \frac{\lambda^{y_{t}}}{y_{t}!}exp(-\lambda)$.

Across the time series, the conditional likelihood is $L(a_{1},a_{2} | y_{t}, y_{t+1}) =\prod_{i = 2}^{7} Pr(y_{i}| y_{i-1}; a_{1}, a_{2})$.

Initial imputation uses random draws from a Poisson with mean $\bar{y}$to impute the missing values. The E-M algorithm then maximizes over this ‘full’ data set and updates the missing values based on model predictions.

We saw only a few participants having genuine missing data in their intrusive memory diaries during baseline (run-in) week, week 4 and week 8. Two participants (one on immediate arm, and one on delayed arm) had one value in their week 4 daily IM diary, and one participant (on delayed arm) had one value in their week 8 daily IM diary, imputed using the method detailed above.

**Multiple Imputation**

The missing data method described above was only used to impute any missing daily IM dairy days, if there was at least one day filled out.

For cases where all 7-days of the daily IM diary was missing, and for other missing values in analysis of secondary outcomes, the multiple imputation procedure was used to impute any missing values with an analytical model. For each outcome, 20 imputations (completed datasets) were generated under missing at random (MAR) assumption. Stata (version 17), and additionally Blimp (version 3.0) were used to impute missingness where appropriate.

- 1. **Sensitivity Analyses**

In addition to the analysis of the intention-to-treat (ITT) population, four sensitivity analyses were conducted to evaluate the robustness of treatment effect estimates across various statistical scenarios, described below.

1. **Observed data only**

Observed data was analysed with the ZINB model.

1. **Observation-level random effects (OLRE) Poisson model**

In addition, observation-level random effects (OLRE) by Multilevel Poisson was used to quantify treatment effect on primary outcomes, using fully imputed data. The OLRE Poisson model includes a random intercept (treat the participant number as a random effect). The model is detailed below where $\gamma_{0i}$ is the random effect for the intercept for each participant i that accounts for the participant-specific variation in the primary endpoint. The random-effects intercepts $\gamma_{0i}$ are drawn from a normal distribution with mean 0 and variance $\sigma_{\gamma_{0}}^{2}$ which is estimated by the model.

$$log\left( \mu_{i} \right)=\alpha_{i}+{ARM}_{i}\beta_{1}+{Baseline}_{i}\beta_{2}$$

$$\alpha_{i} = \alpha+ \gamma_{0i}$$

$$\gamma_{0i}\sim N\left( 0,\sigma_{\gamma_{0}}^{2} \right)$$

1. **Excluding identified outliers**

Outliers were identified through inspection of residual plots and Cook’s distance vs leverage plots using imputed data, and the primary analysis model (ZINB) was then repeated excluding identified outliers (see table of identified outliers below). For the primary outcome were 4 outliers in the intervention condition and 5 in the delayed condition. Normal Poisson (with covariates arm and baseline) was used to detect outliers using daily diary imputed data, where Cook’s distance >1 or abs(residual)>3 was defined as an outlier. Note that multiple imputation is a random process and thereby may generate varying numerical results.

**Table of Identified Outliers**

| **Arm** | **Cooks distance** | **Residuals** | **Primary outcome: Number of intrusive memories in week 4 (total imputed)** |
| --- | --- | --- | --- |
| Immediate | .39225 | -3.336723 | 0 |
| Immediate | .0483475 | 3.381397 | 14 |
| Delayed | .0762115 | 5.153706 | 32 |
| Immediate | 1.677477 | 25.08978 | 61 |
| Immediate | .138473 | 8.654055 | 21 |
| Delayed | .1362478 | 4.655577 | 37 |
| Delayed | .0908845 | 3.384748 | 33 |
| Delayed | 23.60159 | -2.508869 | 36 |
| Delayed | .0293949 | 3.037232 | 26 (after multiple imputation as did not complete any day of primary outcome) |

1. **Per-protocol population**

Finally, the primary analysis model was conducted on the per protocol population only. The per-protocol analysis (PPA) set consisted of participants for whom there were no significant adherence or protocol deviations according to the definitions below:

1. Non-completion of the primary outcome measure
2. Non-completion of guided intervention session/non-adherence to the intervention (see below)

***Definition of adherence to the intervention:***During the first guided session, the participant completes all key components of the intervention including: accurately identifying and briefly listing intrusive memories; choosing an intrusive memory to target; *briefly* bringing to mind the intrusive memory image before gameplay (sufficiently clearly but not so much it becomes overly upsetting); *then* sufficient uninterrupted Tetris game play (c.20 min in total; range 15-25 min); during gameplay actively using mental rotation. Non-adherence may be
indexed by in session reports or behaviours that are incompatible with the steps above e.g. deliberately bringing the memory to mind repeatedly during game play. Technical problems which were resolved in some way, such as not being able to view the video instructions for steps in the study procedure (and instead reading the written transcript), intervention glitches or internet connection problems which do not disturb prevent key components from being completed are not considered protocol deviations. Similarly, minor interruptions during gameplay which do not disturb overall
engagement in the game will not be considered a protocol deviation). In cases where adherence was unclear, a case discussion was held.

Protocol deviations are summarised below. The per protocol analysis consisted of all randomised participants excluding those listed below.

**Table of Protocol Deviations**

| **Arm** | **Reason(s) for deviation** |
| --- | --- |
| Immediate | Completed guided intervention session but did not adhere to intervention |
| Immediate | Did not complete primary outcome |
| Immediate | Did not complete any components of guided intervention session Did not complete primary outcome |
| Immediate | Did not complete primary outcome |
| Immediate | Did not complete primary outcome |
| Immediate | Did not complete primary outcome |
| Immediate | Did not complete any components of guided intervention session Did not complete primary outcome |
| Immediate | Did not complete any components of guided intervention session Did not complete primary outcome |
| Immediate | Completed guided intervention session but did not adhere to intervention |
| Delayed | Did not complete any components of guided intervention session Did not complete primary outcome |
| Delayed | Did not complete any components of guided intervention session Did not complete primary outcome |
| Delayed | Did not complete any components of guided intervention session Did not complete primary outcome |
| Delayed | Did not complete any components of guided intervention session Did not complete primary outcome |

Figure 1 graphically summarises the results of the sensitivity analyses, to evaluate the robustness of treatment effect estimates across various scenarios.


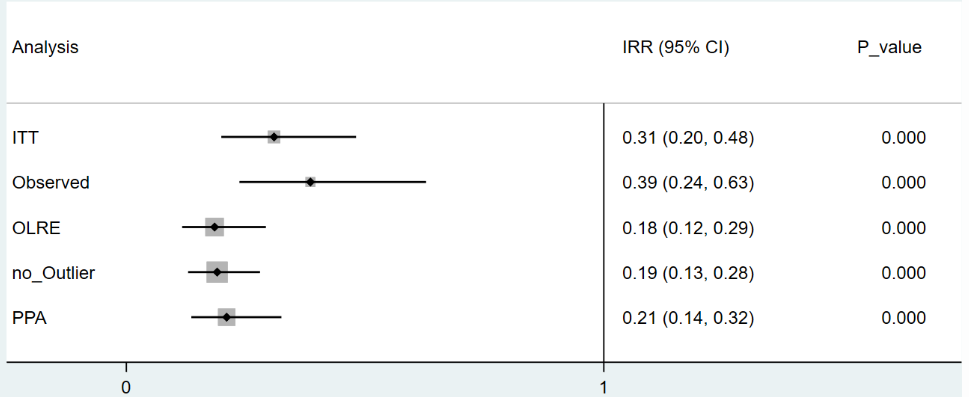


ITT = intention to treat analysis

Observed = analysis of observed data only

OLRE = observation-level random effects

No_Outlier = analysis excluding identified outliers

PPA = analysis of per protocol population

**Supplementary Figure 1**: Results of the sensitivity analyses, evaluating the robustness of treatment effect estimates across various scenarios.

All four sensitivity analyses of the primary outcome showed a significant difference in the number of intrusive memories between the immediate and delayed intervention arm in the same direction as the ITT analysis, indicating robustness of the treatment effect across different scenarios.

1. **SUPPLEMENTARY TABLES**

Supplementary Tables 1 through 8 are provided as a separate electronic file (word).

1. **REFERENCES FOR SUPPLEMENTARY MATERIALS**

1. Agency EM. Guideline on adjustment for baseline covariates in clinical trials. 2013.

2. Vickers AJ, Altman DG. Statistics Notes: Analysing controlled trials with baseline and follow up measurements. BMJ. 2001;323.

3. Manning WG, Mullahy J. Estimating log models: To transform or not to transform? J Health Econ. 2001;20.

4. Ives AR. For testing the significance of regression coefficients, go ahead and log-transform count data. Methods Ecol Evol. 2015;6.

5. Curran-Everett D. Explorations in statistics: The log transformation. Adv Physiol Educ. 2018;42.

6. Chatfield Chris, A. J. Collins. & Bozorgnia AF. The analysis of time series: an introduction. Sixth Edit. New York: Chapman and hall/CRC; 2003.

7. Dempster AP, Laird NM, Rubin DB. Maximum Likelihood from Incomplete Data Via the EM Algorithm. J R Stat Soc Ser B. 1977;39:1–22.
