# Supplementary material for: Reducing intrusive memories after trauma via an imagery-competing task intervention in COVID-19 intensive care staff: a randomised controlled trial
Source: Transl Psychiatry. 2023 Sep 1;13:290. doi: 10.1038/s41398-023-02578-0 (PMC10474101; doi:10.1038/s41398-023-02578-0)
Supplement: Supplementary file 2 — Supplementary Tables [file 41398_2023_2578_MOESM2_ESM.docx]

**Supplementary Table 1.**

*Self-report accuracy ratings of intrusive memory diary completion (primary outcome at week 4)*

|  | Delayed Arm | | | | | Immediate Arm | | | | | All Participants (Combined) | | | | | |
| --- | --- | --- | --- | --- | --- | --- | --- | --- | --- | --- | --- | --- | --- | --- | --- | --- |
|  | n | Mean | SD | Median | IQR | n | Mean | SD | Median | IQR | n | Mean | SD | Median | IQR |  |
| Self-report accuracy ratings of intrusive memory diary completion | | | | | | | | | | | | | | | | |
| Baseline | 39 | 8.21 | 1.47 | 8.0 | 8.0 - 9.0 | 37 | 8.08 | 1.36 | 8.0 | 7.0 - 9.0 | 76 | 8.14 | 1.41 | 8.0 | 7.0 - 9.0 |  |
| Week 4 | 38 | 7.76 | 1.92 | 8.0 | 7.0 - 9.0 | 35 | 8.31 | 2.27 | 9.0 | 8.0 - 10.0 | 73 | 8.03 | 2.10 | 8.0 | 7.0 - 10.0 |  |
| Week 8 | 29 | 8.17 | 1.39 | 8.0 | 7.0 - 9.0 |  |  |  |  |  |  |  |  |  |  |  |
| *Note.* The intrusive memory diary was completed at week 8 only by participants in the delayed intervention arm. The self-reported accuracy is rated on 11-point scale from 0 (not accurately at all) to 10 (extremely accurately).  SD = Standard Deviation; IQR = Interquartile Range. | | | | | | | | | | | | | | | | |

**Supplementary Table 2.**

*Between-group analysis for Ordinal Variables of Secondary Outcomes*

|  | Delayed Arm | | | Immediate Arm | | | ITT Treatment Effect Estimate | | |
| --- | --- | --- | --- | --- | --- | --- | --- | --- | --- |
|  | n | Freq. | % | n | Freq. | % | Odds Ratio | 95% CI | |
| **Intrusive Memory Ratings** |  |  |  |  |  |  |  |  | |
| Approximately how often did intrusive memories of the traumatic event pop into your mind? |  |  |  |  |  |  |  |  | |
| Baseline | 43 |  |  | 42 |  |  |  |  | |
| *Never* |  | 0 | 0 |  | 0 | 0 |  |  | |
| *Once* |  | 0 | 0 |  | 1 | 2.4 |  |  | |
| *Twice* |  | 1 | 2.3 |  | 0 | 0 |  |  | |
| *Every other day* |  | 14 | 32.6 |  | 6 | 14.3 |  |  | |
| *Once a day* |  | 7 | 16.3 |  | 6 | 14.3 |  |  | |
| *Several times a day* |  | 18 | 41.9 |  | 28 | 66.7 |  |  | |
| *Many times a day* |  | 3 | 7.0 |  | 1 | 2.4 |  |  | |
| Week 4 | 38 |  |  | 35 |  |  | 0.02*** | (0.00, 0.13) | |
| *Never* |  | 0 | 0 |  | 11 | 31.4 |  |  | |
| *Once* |  | 1 | 2.6 |  | 6 | 17.1 |  |  | |
| *Twice* |  | 2 | 5.3 |  | 5 | 14.3 |  |  | |
| *Every other day* |  | 10 | 26.3 |  | 6 | 17.1 |  |  | |
| *Once a day* |  | 7 | 18.4 |  | 4 | 11.4 |  |  | |
| *Several times a day* |  | 15 | 39.5 |  | 2 | 5.7 |  |  | |
| *Many times a day* |  | 3 | 7.9 |  | 1 | 2.9 |  |  | |
| Each time, for (approximately) how long did they interfere with what you were doing? | |  |  |  |  |  |  |  | |
| Baseline | 43 |  |  | 42 |  |  |  |  | |
| *<1min* |  | 17 | 39.5 |  | 16 | 38.1 |  |  | |
| *1-5mins* |  | 21 | 48.8 |  | 17 | 40.5 |  |  | |
| *6-10mins* |  | 3 | 7.0 |  | 9 | 21.4 |  |  | |
| *11-30mins* |  | 1 | 2.3 |  | 0 | 0 |  |  | |
| *31-60mins* |  | 1 | 2.3 |  | 0 | 0 |  |  | |
| Week 4 | 38 |  |  | 35 |  |  | 0.03** | (0.00, 0.35) | |
| *<1min* |  | 15 | 39.5 |  | 27 | 77.1 |  |  | |
| *1-5mins* |  | 19 | 50.0 |  | 8 | 22.9 |  |  | |
| *6-10mins* |  | 3 | 7.9 |  | 0 | 0 |  |  | |
| *11-30mins* |  | 1 | 2.6 |  | 0 | 0 |  |  | |
| *31-60mins* |  | 0 | 0 |  | 0 | 0 |  |  | |
| **Wellbeing (EQ-5D-5L)** |  |  |  |  |  |  |  |  | |
| EQ-5D-5L Mobility Subscale |  |  |  |  |  |  |  |  | |
| Baseline | 42 |  |  | 40 |  |  |  |  | |
| *I have no problems in walking about* |  | 36 | 85.7 |  | 33 | 82.5 |  |  | |
| *I have slight problems in walking about* |  | 4 | 9.5 |  | 5 | 12.5 |  |  | |
| *I have moderate problems in walking about* |  | 1 | 2.4 |  | 2 | 5.0 |  |  | |
| *I have severe problems in walking about* |  | 1 | 2.4 |  | 0 | 0 |  |  | |
| *I am unable to walk about* |  | 0 | 0 |  | 0 | 0 |  |  | |
| Week 4 | 38 |  |  | 28 |  |  | 1.01 | (0.22, 4.64) | |
| *I have no problems in walking about* |  | 34 | 89.5 |  | 25 | 89.3 |  |  | |
| *I have slight problems in walking about* |  | 3 | 7.9 |  | 3 | 10.7 |  |  | |
| *I have moderate problems in walking about* |  | 0 | 0 |  | 0 | 0 |  |  | |
| *I have severe problems in walking about* |  | 1 | 2.6 |  | 0 | 0 |  |  | |
| *I am unable to walk about* |  | 0 | 0 |  | 0 | 0 |  |  | |
| EQ-5D-5L Self-Care Subscale |  |  |  |  |  |  |  |  | |
| Baseline | 42 |  |  | 40 |  |  |  |  | |
| *I have no problems washing or dressing myself* |  | 38 | 90.5 |  | 36 | 90.0 |  |  | |
| *I have slight problems washing or dressing myself* |  | 3 | 7.1 |  | 4 | 10.0 |  |  | |
| *I have moderate problems washing or dressing myself* |  | 0 | 0 |  | 0 | 0 |  |  | |
| *I have severe problems washing or dressing myself* |  | 1 | 2.4 |  | 0 | 0 |  |  | |
| *I am unable to wash or dress myself* |  | 0 | 0 |  | 0 | 0 |  |  | |
| Week 4 | 38 |  |  | 28 |  |  | 0.27 | (0.03, 2.36) | |
| *I have no problems washing or dressing myself* |  | 33 | 86.8 |  | 27 | 96.4 |  |  | |
| *I have slight problems washing or dressing myself* |  | 5 | 13.2 |  | 1 | 3.6 |  |  | |
| *I have moderate problems washing or dressing myself* |  | 0 | 0 |  | 0 | 0 |  |  | |
| *I have severe problems washing or dressing myself* |  | 0 | 0 |  | 0 | 0 |  |  | |
| *I am unable to wash or dress myself* |  | 0 | 0 |  | 0 | 0 |  |  | |
| EQ-5D-5L Usual Activities Subscale | |  |  |  |  |  |  |  | |
| Baseline | 42 |  |  | 40 |  |  |  |  | |
| *I have no problems doing my usual activities* |  | 26 | 61.9 |  | 22 | 55.0 |  |  | |
| *I have slight problems doing my usual activities* |  | 10 | 23.8 |  | 15 | 37.5 |  |  | |
| *I have moderate problems doing my usual activities* |  | 5 | 11.9 |  | 2 | 5.0 |  |  | |
| *I have severe problems doing my usual activities* |  | 0 | 0 |  | 1 | 2.5 |  |  | |
| *I am unable to do my usual activities* |  | 1 | 2.4 |  | 0 | 0 |  |  | |
| Week 4 | 38 |  |  | 28 |  |  | 0.04* | (0.00, 0.70) | |
| *I have no problems doing my usual activities* |  | 23 | 60.5 |  | 24 | 85.7 |  |  | |
| *I have slight problems doing my usual activities* |  | 10 | 26.3 |  | 4 | 14.3 |  |  | |
| *I have moderate problems doing my usual activities* |  | 4 | 10.5 |  | 0 | 0 |  |  | |
| *I have severe problems doing my usual activities* |  | 1 | 2.6 |  | 0 | 0 |  |  | |
| *I am unable to do my usual activities* |  | 0 | 0 |  | 0 | 0 |  |  | |
| EQ-5D-5L Pain / Discomfort Subscale | |  |  |  |  |  |  |  | |
| Baseline | 42 |  |  | 40 |  |  |  |  | |
| *I have no pain or discomfort* |  | 16 | 38.1 |  | 15 | 37.5 |  |  | |
| *I have slight pain or discomfort* |  | 20 | 47.6 |  | 19 | 47.5 |  |  | |
| *I have moderate pain or discomfort* |  | 5 | 11.9 |  | 5 | 12.5 |  |  | |
| *I have severe pain or discomfort* |  | 0 | 0 |  | 1 | 2.5 |  |  | |
| *I have extreme pain or discomfort* |  | 1 | 2.4 |  | 0 | 0 |  |  | |
| Week 4 | 38 |  |  | 28 |  |  | 0.74 | (0.29, 1.86) | |
| *I have no pain or discomfort* |  | 14 | 36.8 |  | 15 | 53.6 |  |  | |
| *I have slight pain or discomfort* |  | 19 | 50.0 |  | 9 | 32.1 |  |  | |
| *I have moderate pain or discomfort* |  | 4 | 10.5 |  | 4 | 14.3 |  |  | |
| *I have severe pain or discomfort* |  | 1 | 2.6 |  | 0 | 0 |  |  | |
| *I have extreme pain or discomfort* |  | 0 | 0 |  | 0 | 0 |  |  | |
| EQ-5D-5L Anxiety / Depression Subscale | |  |  |  |  |  |  |  | |
| Baseline | 42 |  |  | 40 |  |  |  |  | |
| *I am not anxious or depressed* |  | 4 | 9.5 |  | 4 | 10.0 |  |  | |
| *I am slightly anxious or depressed* |  | 14 | 33.3 |  | 11 | 27.5 |  |  | |
| *I am moderately anxious or depressed* |  | 21 | 50.0 |  | 18 | 45.0 |  |  | |
| *I am severely anxious or depressed* |  | 3 | 7.1 |  | 5 | 12.5 |  |  | |
| *I am extremely anxious or depressed* |  | 0 | 0 |  | 2 | 5.0 |  |  | |
| Week 4 | 38 |  |  | 28 |  |  | 0.24* | (0.06, 0.97) | |
| *I am not anxious or depressed* |  | 5 | 13.2 |  | 12 | 42.9 |  |  | |
| *I am slightly anxious or depressed* |  | 17 | 44.7 |  | 11 | 39.3 |  |  | |
| *I am moderately anxious or depressed* |  | 14 | 36.8 |  | 3 | 10.7 |  |  | |
| *I am severely anxious or depressed* |  | 1 | 2.6 |  | 2 | 7.1 |  |  | |
| *I am extremely anxious or depressed* |  | 1 | 2.6 |  | 0 | 0 |  |  | |
| *Notes.* n = total number observed; Freq. = Frequency of ordinal response items; CI = Confidence Interval; EQ-5D-5L = European Quality of Life Five Dimension Five Level Scale. ****p* < .001; ** *p* <.01; **p* < .05 | | | | | | | | |  |

**Supplementary Table 3.**

*Within-Group Analysis of Secondary Outcomes*

|  | Pre-Intervention | | | | | Post-Intervention | | | | | ITT Treatment Effect Estimate | |
| --- | --- | --- | --- | --- | --- | --- | --- | --- | --- | --- | --- | --- |
| **Number of Intrusive Memories** |  |  |  |  |  |  |  |  |  |  |  |  |
|  | n | Mean | SD | Median | IQR | n | Mean | SD | Median | IQR | Incidence Rate Ratio | 95% CI |
| Number of intrusive memories recorded during week |  |  |  |  |  |  |  |  |  |  |  |  |
| Immediate (Baseline, Week 4) | 36 | 18.50 | 13.61 | 14.50 | 10.0 - 21.5 | 36 | 4.03 | 10.68 | 1.00 | 0.0 - 3.0 | 0.22*** | (0.10, 0.45) |
| Delayed Arm (Week 4, Week 8) | 39 | 12.46 | 9.28 | 10.00 | 6.0 - 17.0 | 32 | 3.78 | 7.23 | 1.00 | 0.0 - 2.5 | 0.31*** | (0.21, 0.45) |
|  |  |  |  |  |  |  |  |  |  |  |  |  |
| **Impact of Intrusive Memories** |  |  |  |  |  |  |  |  |  |  |  |  |
|  | n | Freq. | % |  |  | n | Freq. | % |  |  | Odds Ratio | 95% CI |
| Approximately how often did intrusive memories of the traumatic event pop into your mind? How many times per day? |  |  |  |  |  |  |  |  |  |  |  |  |
| Immediate Arm (Baseline, Week 4) | 42 |  |  |  |  | 35 |  |  |  |  | 0.01*** | (0.00, 0.04) |
| *Never* |  | 0 | 0.0 |  |  |  | 11 | 31.4 |  |  |  |  |
| *Once* |  | 1 | 2.4 |  |  |  | 6 | 17.1 |  |  |  |  |
| *Twice* |  | 0 | 0.0 |  |  |  | 5 | 14.3 |  |  |  |  |
| *Every other day* |  | 6 | 14.3 |  |  |  | 6 | 17.1 |  |  |  |  |
| *Once a day* |  | 6 | 14.3 |  |  |  | 4 | 11.4 |  |  |  |  |
| *Several times a day* |  | 28 | 66.7 |  |  |  | 2 | 5.7 |  |  |  |  |
| *Many times a day* |  | 1 | 2.4 |  |  |  | 1 | 2.9 |  |  |  |  |
| Immediate Arm (Baseline, Week 8) | 42 |  |  |  |  | 31 |  |  |  |  | 0.00*** | (0.00, 0.01) |
| *Never* |  | 0 | 0.0 |  |  |  | 14 | 45.2 |  |  |  |  |
| *Once* |  | 1 | 2.4 |  |  |  | 3 | 9.7 |  |  |  |  |
| *Twice* |  | 0 | 0.0 |  |  |  | 7 | 22.6 |  |  |  |  |
| *Every other day* |  | 6 | 14.3 |  |  |  | 5 | 16.1 |  |  |  |  |
| *Once a day* |  | 6 | 14.3 |  |  |  | 0 | 0.0 |  |  |  |  |
| *Several times a day* |  | 28 | 66.7 |  |  |  | 2 | 6.5 |  |  |  |  |
| *Many times a day* |  | 1 | 2.4 |  |  |  | 0 | 0.0 |  |  |  |  |
| Delayed Arm (Week 4, Week 8) | 38 |  |  |  |  | 30 |  |  |  |  | 0.02*** | (0.01, 0.06) |
| *Never* |  | 0 | 0.0 |  |  |  | 7 | 23.3 |  |  |  |  |
| *Once* |  | 1 | 2.6 |  |  |  | 12 | 40.0 |  |  |  |  |
| *Twice* |  | 2 | 5.3 |  |  |  | 3 | 10.0 |  |  |  |  |
| *Every other day* |  | 10 | 26.3 |  |  |  | 4 | 13.3 |  |  |  |  |
| *Once a day* |  | 7 | 18.4 |  |  |  | 1 | 3.3 |  |  |  |  |
| *Several times a day* |  | 15 | 39.5 |  |  |  | 2 | 6.7 |  |  |  |  |
| *Many times a day* |  | 3 | 7.9 |  |  |  | 1 | 3.3 |  |  |  |  |
|  |  |  |  |  |  |  |  |  |  |  |  |  |
|  | n | Mean | SD | Median | IQR | n | Mean | SD | Median | IQR | Mean Difference | 95% CI |
| How distressing were your intrusive memories?^1^ |  |  |  |  |  |  |  |  |  |  |  |  |
| Immediate Arm (Baseline, Week 4) | 42 | 6.02 | 1.83 | 6.00 | 5.0 - 7.0 | 35 | 2.86 | 2.45 | 3.00 | 0.0 - 5.0 | -3.11*** | (-3.88, -2.34) |
| Immediate Arm (Baseline, Week 8) | 42 | 6.02 | 1.83 | 6.00 | 5.0 - 7.0 | 31 | 2.00 | 2.44 | 2.00 | 0.0 - 3.0 | -3.90*** | (-4.66, -3.14) |
| Delayed Arm (Week 4, Week 8) | 38 | 5.53 | 1.94 | 6.00 | 4.0 - 7.0 | 30 | 2.87 | 2.67 | 2.00 | 1.0 - 5.0 | -2.57*** | (-3.53, -1.61) |
| How much did they disrupt your concentration?^1^ |  |  |  |  |  |  |  |  |  |  |  |  |
| Immediate Arm (Baseline, Week 4) | 42 | 7.05 | 2.29 | 7.50 | 6.0 - 9.0 | 35 | 2.74 | 2.76 | 3.00 | 0.0 - 5.0 | -4.11*** | (-4.86, -3.36) |
| Immediate Arm (Baseline, Week 8) | 42 | 7.05 | 2.29 | 7.50 | 6.0 - 9.0 | 31 | 1.97 | 2.40 | 2.00 | 0.0 - 3.0 | -4.81*** | (-5.56, -4.07) |
| Delayed Arm (Week 4, Week 8) | 38 | 5.95 | 1.72 | 6.50 | 5.0 - 7.0 | 30 | 2.67 | 2.19 | 2.50 | 0.0 - 5.0 | -3.15*** | (-4.00, -2.31) |
| How much did they interfere with what you were doing?^1^ |  |  |  |  |  |  |  |  |  |  |  |  |
| Immediate Arm (Baseline, Week 4) | 42 | 5.95 | 2.26 | 6.00 | 5.0 - 8.0 | 35 | 2.26 | 2.32 | 3.00 | 0.0 - 3.0 | -3.44*** | (-4.12, -2.76) |
| Immediate Arm (Baseline, Week 8) | 42 | 5.95 | 2.26 | 6.00 | 5.0 - 8.0 | 31 | 1.55 | 2.01 | 1.00 | 0.0 - 2.0 | -4.05*** | (-4.73, -3.36) |
| Delayed Arm (Week 4, Week 8) | 38 | 5.13 | 1.99 | 5.00 | 4.0 - 6.0 | 30 | 2.27 | 2.08 | 1.50 | 1.0 - 4.0 | -2.79*** | (-3.63, -1.95) |
|  |  |  |  |  |  |  |  |  |  |  |  |  |
|  | n | Freq. | % |  |  | n | Freq. | % |  |  | Odds Ratio | 95% CI |
| Each time, for (approximately) how long did they interfere with what you were doing? |  |  |  |  |  |  |  |  |  |  |  |  |
| Immediate Arm (Baseline, Week 4) | 42 |  |  |  |  | 35 |  |  |  |  | 0.04*** | (0.01, 0.18) |
| *<1min* |  | 16 | 38.1 |  |  |  | 27 | 77.1 |  |  |  |  |
| *1-5mins* |  | 17 | 40.5 |  |  |  | 8 | 22.9 |  |  |  |  |
| *6-10mins* |  | 9 | 21.4 |  |  |  | 0 | 0.0 |  |  |  |  |
| *11-30mins* |  | 0 | 0.0 |  |  |  | 0 | 0.0 |  |  |  |  |
| *31-60mins* |  | 0 | 0.0 |  |  |  | 0 | 0.0 |  |  |  |  |
| Immediate Arm (Baseline, Week 8) | 42 |  |  |  |  |  |  |  |  |  | 0.02*** | (0.00, 0.09) |
| *<1min* |  | 16 | 38.1 |  |  |  | 27 | 87.1 |  |  |  |  |
| *1-5mins* |  | 17 | 40.5 |  |  |  | 4 | 12.9 |  |  |  |  |
| *6-10mins* |  | 9 | 21.4 |  |  |  | 0 | 0.0 |  |  |  |  |
| *11-30mins* |  | 0 | 0.0 |  |  |  | 0 | 0.0 |  |  |  |  |
| *31-60mins* |  | 0 | 0.0 |  |  |  | 0 | 0.0 |  |  |  |  |
| Delayed Arm (Week 4, Week 8) | 38 |  |  |  |  | 30 |  |  |  |  | 0.10** | (0.03, 0.41) |
| *<1min* |  | 15 | 39.5 |  |  |  | 23 | 76.7 |  |  |  |  |
| *1-5mins* |  | 19 | 50.0 |  |  |  | 4 | 13.3 |  |  |  |  |
| *6-10mins* |  | 3 | 7.9 |  |  |  | 2 | 6.7 |  |  |  |  |
| *11-30mins* |  | 1 | 2.6 |  |  |  | 1 | 3.3 |  |  |  |  |
| *31-60mins* |  | 0 | 0.0 |  |  |  | 0 | 0.0 |  |  |  |  |
|  |  |  |  |  |  |  |  |  |  |  |  |  |
|  | n | Mean | SD | Median | IQR | n | Mean | SD | Median | IQR | Mean Difference | 95% CI |
| How much did your intrusive memories affect your work functioning?^1^ |  |  |  |  |  |  |  |  |  |  |  |  |
| Immediate Arm (Baseline, Week 4) | 42 | 4.71 | 2.64 | 5.00 | 3.0 - 6.0 | 35 | 1.71 | 2.63 | 0.00 | 0.0 - 3.0 | -2.90*** | (-3.65, -2.15) |
| Immediate Arm (Baseline, Week 8) | 42 | 4.71 | 2.64 | 5.00 | 3.0 - 6.0 | 31 | 1.00 | 1.65 | 0.00 | 0.0 - 2.0 | -3.62*** | (-4.37, -2.88) |
| Delayed Arm (Week 4, Week 8) | 38 | 3.97 | 2.81 | 4.00 | 2.0 - 6.0 | 30 | 1.93 | 2.27 | 1.00 | 0.0 - 4.0 | -1.99*** | (-2.92, -1.05) |
| How much did your intrusive memories affect your functioning in other areas of your life?^1^ |  |  |  |  |  |  |  |  |  |  |  |  |
| Immediate Arm (Baseline, Week 4) | 42 | 5.55 | 2.21 | 6.00 | 4.0 - 7.0 | 35 | 1.89 | 2.35 | 2.00 | 0.0 - 3.0 | -3.55*** | (-4.31, -2.79) |
| Immediate Arm (Baseline, Week 8) | 42 | 5.55 | 2.21 | 6.00 | 4.0 - 7.0 | 31 | 1.58 | 2.57 | 0.00 | 0.0 - 3.0 | -3.75*** | (-4.52, -2.99) |
| Delayed Arm (Week 4, Week 8) | 38 | 4.42 | 2.78 | 4.50 | 2.0 - 7.0 | 30 | 1.53 | 1.83 | 1.00 | 0.0 - 3.0 | -2.80*** | (-3.78, -1.81) |
|  |  |  |  |  |  |  |  |  |  |  |  |  |
| **Clinical Symptoms** |  |  |  |  |  |  |  |  |  |  |  |  |
|  | n | Mean | SD | Median | IQR | n | Mean | SD | Median | IQR | Mean Difference | 95% CI |
| PTSD symptoms (PCL-5) |  |  |  |  |  |  |  |  |  |  |  |  |
| Immediate Arm (Baseline, Week 4) | 42 | 8.71 | 3.64 | 8.00 | 5.0 - 12.0 | 31 | 4.26 | 2.86 | 4.00 | 2.0 - 6.0 | -3.85*** | (-4.72, -2.97) |
| Immediate Arm (Baseline, Week 8) | 42 | 8.71 | 3.64 | 8.00 | 5.0 - 12.0 | 31 | 2.35 | 3.13 | 1.00 | 0.0 - 3.0 | -5.92*** | (-6.86, -4.98) |
| Delayed Arm (Week 4, Week 8) | 38 | 6.74 | 2.62 | 6.00 | 5.0 - 9.0 | 31 | 2.87 | 2.53 | 2.00 | 1.0 - 5.0 | -3.77*** | (-4.63, -2.92) |
| Insomnia (SCI-08) |  |  |  |  |  |  |  |  |  |  |  |  |
| Immediate Arm (Baseline, Week 4) | 41 | 12.37 | 7.75 | 11.00 | 7.0 - 16.0 | 30 | 19.13 | 8.27 | 20.50 | 14.0 - 26.0 | 5.77*** | (3.53, 8.01) |
| Immediate Arm (Baseline, Week 8) | 41 | 12.37 | 7.75 | 11.00 | 7.0 - 16.0 | 31 | 20.65 | 7.75 | 20.00 | 13.0 - 27.0 | 7.34*** | (5.17, 9.51) |
| Delayed Arm (Week 4, Week 8) | 38 | 12.71 | 6.06 | 12.00 | 10.0 - 16.0 | 31 | 19.23 | 7.55 | 19.00 | 15.0 - 25.0 | 6.16*** | (4.34, 7.97) |
| Anxiety Symptoms (GAD-2) |  |  |  |  |  |  |  |  |  |  |  |  |
| Immediate Arm (Baseline, Week 4) | 41 | 3.83 | 1.80 | 4.00 | 3.0 - 5.0 | 30 | 2.07 | 1.91 | 2.00 | 0.0 - 3.0 | -1.39*** | (-1.99, -0.79) |
| Immediate Arm (Baseline, Week 8) | 41 | 3.83 | 1.80 | 4.00 | 3.0 - 5.0 | 31 | 1.65 | 1.98 | 1.00 | 0.0 - 2.0 | -1.79*** | (-2.35, -1.22) |
| Delayed Arm (Week 4, Week 8) | 38 | 2.84 | 1.79 | 2.00 | 2.0 - 4.0 | 31 | 1.29 | 1.55 | 1.00 | 0.0 - 2.0 | -1.55*** | (-2.10, -1.00) |
| Depression Symptoms (PHQ-2) |  |  |  |  |  |  |  |  |  |  |  |  |
| Immediate Arm (Baseline, Week 4) | 41 | 2.76 | 1.48 | 2.00 | 2.0 - 4.0 | 30 | 1.53 | 1.50 | 1.00 | 0.0 - 2.0 | -0.97** | (-1.53, -0.41) |
| Immediate Arm (Baseline, Week 8) | 41 | 2.76 | 1.48 | 2.00 | 2.0 - 4.0 | 31 | 1.23 | 1.61 | 1.00 | 0.0 - 2.0 | -1.22*** | (-1.76, -0.69) |
| Delayed Arm (Week 4, Week 8) | 38 | 1.92 | 1.62 | 2.00 | 1.0 - 2.0 | 31 | 1.23 | 1.87 | 0.00 | 0.0 - 2.0 | -0.71** | (-1.22, -0.20) |
|  |  |  |  |  |  |  |  |  |  |  |  |  |
| **Post-Trauma Distress, Impact of Events Scale** |  |  |  |  |  |  |  |  |  |  |  |  |
|  | n | Mean | SD | Median | IQR | n | Mean | SD | Median | IQR | Mean Difference | 95% CI |
| Impact of Events Scale Total |  |  |  |  |  |  |  |  |  |  |  |  |
| Immediate Arm (Baseline, Week 4) | 41 | 2.24 | 0.77 | 2.28 | 1.82 - 2.91 | 32 | 0.83 | 0.58 | 0.82 | 0.36 - 1.23 | -1.31*** | (-1.52, -1.10) |
| Immediate Arm (Baseline, Week 8) | 41 | 2.24 | 0.77 | 2.28 | 1.82 - 2.91 | 31 | 0.58 | 0.59 | 0.50 | 0.10 - 0.82 | -1.59*** | (-1.80, -1.38) |
| Delayed Arm (Week 4, Week 8) | 38 | 1.70 | 0.56 | 1.67 | 1.41 - 2.05 | 32 | 0.78 | 0.66 | 0.58 | 0.33 - 1.07 | -0.90*** | (-1.07, -0.73) |
| Impact of Events, Intrusion subscale |  |  |  |  |  |  |  |  |  |  |  |  |
| Immediate Arm (Baseline, Week 4) | 41 | 2.52 | 0.91 | 2.43 | 2.0 - 3.29 | 32 | 0.87 | 0.63 | 0.86 | 0.29 - 1.43 | -1.56*** | (-1.81, -1.31) |
| Immediate Arm (Baseline, Week 8) | 41 | 2.52 | 0.91 | 2.43 | 2.0 - 3.29 | 31 | 0.62 | 0.62 | 0.58 | 0.0 - 0.86 | -1.84*** | (-2.09, -1.59) |
| Delayed Arm (Week 4, Week 8) | 38 | 1.86 | 0.67 | 1.86 | 1.43 - 2.29 | 32 | 0.85 | 0.71 | 0.72 | 0.43 - 1.0 | -0.99*** | (-1.20, -0.78) |
| Impact of Events, Avoidance subscale |  |  |  |  |  |  |  |  |  |  |  |  |
| Immediate Arm (Baseline, Week 4) | 41 | 2.19 | 0.81 | 2.13 | 1.63 - 2.75 | 32 | 1.06 | 0.75 | 1.00 | 0.56 - 1.50 | -1.06*** | (-1.31, -0.80) |
| Immediate Arm (Baseline, Week 8) | 41 | 2.19 | 0.81 | 2.13 | 1.63 - 2.75 | 31 | 0.68 | 0.76 | 0.50 | 0.0 - 1.13 | -1.45*** | (-1.71, -1.18) |
| Delayed Arm (Week 4, Week 8) | 38 | 1.95 | 0.70 | 1.75 | 1.50 - 2.50 | 32 | 0.87 | 0.83 | 0.50 | 0.19 - 1.31 | -1.08*** | (-1.32, -0.85) |
| Impact of Events, Hyperarousal subscale |  |  |  |  |  |  |  |  |  |  |  |  |
| Immediate Arm (Baseline, Week 4) | 41 | 2.03 | 0.96 | 2.15 | 1.29 - 2.72 | 32 | 0.53 | 0.52 | 0.36 | 0.08 - 0.79 | -1.34*** | (-1.55, -1.14) |
| Immediate Arm (Baseline, Week 8) | 41 | 2.03 | 0.96 | 2.15 | 1.29 - 2.72 | 31 | 0.41 | 0.55 | 0.29 | 0.0 - 0.43 | -1.49*** | (-1.70, -1.28) |
| Delayed Arm (Week 4, Week 8) | 38 | 1.27 | 0.68 | 1.29 | 0.86 - 1.58 | 32 | 0.60 | 0.63 | 0.43 | 0.15 - 0.79 | -0.61*** | (-0.78, -0.44) |
|  |  |  |  |  |  |  |  |  |  |  |  |  |
| **Work Functioning** |  |  |  |  |  |  |  |  |  |  |  |  |
|  | n | Mean | SD | Median | IQR | n | Mean | SD | Median | IQR | Mean Difference | 95% CI |
| Work Engagement (SWEBO) |  |  |  |  |  |  |  |  |  |  |  |  |
| Immediate Arm (Baseline, Week 4) | 40 | 2.03 | 0.46 | 1.95 | 1.75 - 2.25 | 28 | 2.59 | 0.66 | 2.70 | 2.25 - 3.0 | 0.43*** | (0.24, 0.61) |
| Immediate Arm (Baseline, Week 8) | 40 | 2.03 | 0.46 | 1.95 | 1.75 - 2.25 | 31 | 2.66 | 0.69 | 2.60 | 2.0 - 3.10 | 0.56*** | (0.35, 0.77) |
| Delayed Arm (Week 4, Week 8) | 38 | 2.10 | 0.48 | 2.05 | 1.70 - 2.50 | 31 | 2.50 | 0.62 | 2.60 | 2.0 - 3.0 | 0.39** | (0.17, 0.61) |
| Work Burnout (SWEBO) |  |  |  |  |  |  |  |  |  |  |  |  |
| Immediate Arm (Baseline, Week 4) | 40 | 2.45 | 0.54 | 2.39 | 2.12 - 2.78 | 28 | 1.80 | 0.54 | 1.78 | 1.40 - 2.17 | -0.57*** | (-0.77, -0.38) |
| Immediate Arm (Baseline, Week 8) | 40 | 2.45 | 0.54 | 2.39 | 2.12 - 2.78 | 31 | 1.82 | 0.56 | 1.89 | 1.34 - 2.12 | -0.55*** | (-0.74, -0.36) |
| Delayed Arm (Week 4, Week 8) | 38 | 2.40 | 0.61 | 2.45 | 2.0 - 2.89 | 31 | 1.97 | 0.61 | 1.89 | 1.45 - 2.45 | -0.43*** | (-0.65, -0.21) |
|  | n | Mean | SD | Median | IQR | n | Mean | SD | Median | IQR | Incidence Rate Ratio | 95% CI |
| Sickness Absence (Number of sick days over past four weeks) |  |  |  |  |  |  |  |  |  |  |  |  |
| Immediate Arm (Baseline, Week 4) | 40 | 3.80 | 6.91 | 0.00 | 0.0 - 4.5 | 28 | 3.79 | 7.36 | 0.00 | 0.0 - 3.0 | 0.99 | (0.55, 1.80) |
| Immediate Arm (Baseline, Week 8) | 40 | 3.80 | 6.91 | 0.00 | 0.0 - 4.5 | 31 | 1.42 | 5.16 | 0.00 | 0.0 - 0.0 | 0.37* | (0.15, 0.94) |
| Delayed Arm (Week 4, Week 8) | 38 | 3.45 | 7.61 | 0.00 | 0.0 - 2.0 | 31 | 1.65 | 5.47 | 0.00 | 0.0 - 0.0 | 0.47 | (0.20, 1.09) |
|  | n | Mean | SD | Median | IQR | n | Mean | SD | Median | IQR | Mean Difference | 95% CI |
| Intention to leave job |  |  |  |  |  |  |  |  |  |  |  |  |
| Immediate Arm (Baseline, Week 4) | 40 | 8.65 | 3.79 | 9.50 | 5.0 - 11.50 | 28 | 9.36 | 4.63 | 9.00 | 4.50 - 13.50 | 0.46 | (-0.53, 1.45) |
| Immediate Arm (Baseline, Week 8) | 40 | 8.65 | 3.79 | 9.50 | 5.0 - 11.50 | 31 | 9.94 | 4.62 | 11.00 | 6.0 - 14.0 | 1.06 | (-0.08, 2.20) |
| Delayed Arm (Week 4, Week 8) | 38 | 10.32 | 3.35 | 11.00 | 8.0 - 13.0 | 31 | 10.26 | 3.86 | 11.00 | 7.0 - 14.0 | 0.02 | (-1.06, 1.10) |
|  |  |  |  |  |  |  |  |  |  |  |  |  |
| **Wellbeing** |  |  |  |  |  |  |  |  |  |  |  |  |
|  | n | Mean | SD | Median | IQR | n | Mean | SD | Median | IQR | Mean Difference | 95% CI |
| WHODAS |  |  |  |  |  |  |  |  |  |  |  |  |
| Immediate Arm (Baseline, Week 4) | 40 | 25.00 | 15.23 | 25.00 | 12.50 - 34.38 | 28 | 13.54 | 11.94 | 10.42 | 3.13 - 20.84 | -10.25*** | (-14.01, -6.49) |
| Immediate Arm (Baseline, Week 8) | 40 | 25.00 | 15.23 | 25.00 | 12.50 - 34.38 | 31 | 10.76 | 10.65 | 8.34 | 0.0 - 18.75 | -12.89*** | (-16.63, -9.16) |
| Delayed Arm (Week 4, Week 8) | 38 | 22.37 | 14.40 | 18.75 | 10.42 - 35.42 | 31 | 11.90 | 14.55 | 6.25 | 0.0 - 22.92 | -10.47*** | (-14.75, -6.18) |
| EQ-5D-5L |  |  |  |  |  |  |  |  |  |  |  |  |
|  | n | Freq. | % |  |  | n | Freq. | % |  |  | Odds Ratio | 95% CI |
| EQ-5D-5L Mobility Subscale |  |  |  |  |  |  |  |  |  |  |  |  |
| Immediate Arm (Baseline, Week 4) | 40 |  |  |  |  | 28 |  |  |  |  | 0.54 | (0.16, 1.89) |
| *I have no problems in walking about* |  | 33 | 82.5 |  |  |  | 25 | 89.3 |  |  |  |  |
| *I have slight problems in walking about* |  | 5 | 12.5 |  |  |  | 3 | 10.7 |  |  |  |  |
| *I have moderate problems in walking about* |  | 2 | 5.0 |  |  |  | 0 | 0.0 |  |  |  |  |
| *I have severe problems in walking about* |  | 0 | 0.0 |  |  |  | 0 | 0.0 |  |  |  |  |
| *I am unable to walk about* |  | 0 | 0.0 |  |  |  | 0 | 0.0 |  |  |  |  |
| Immediate Arm (Baseline, Week 8) | 40 |  |  |  |  | 31 |  |  |  |  | 1.45 | (0.55, 3.87) |
| *I have no problems in walking about* |  | 33 | 82.5 |  |  |  | 23 | 74.2 |  |  |  |  |
| *I have slight problems in walking about* |  | 5 | 12.5 |  |  |  | 7 | 22.6 |  |  |  |  |
| *I have moderate problems in walking about* |  | 2 | 5.0 |  |  |  | 1 | 3.2 |  |  |  |  |
| *I have severe problems in walking about* |  | 0 | 0.0 |  |  |  | 0 | 0.0 |  |  |  |  |
| *I am unable to walk about* |  | 0 | 0.0 |  |  |  | 0 | 0.0 |  |  |  |  |
| Delayed Arm (Week 4, Week 8) | 38 |  |  |  |  | 31 |  |  |  |  | 1.30 | (0.41, 4.11) |
| *I have no problems in walking about* |  | 34 | 89.5 |  |  |  | 27 | 87.1 |  |  |  |  |
| *I have slight problems in walking about* |  | 3 | 7.9 |  |  |  | 1 | 3.2 |  |  |  |  |
| *I have moderate problems in walking about* |  | 0 | 0.0 |  |  |  | 2 | 6.5 |  |  |  |  |
| *I have severe problems in walking about* |  | 1 | 2.6 |  |  |  | 1 | 3.2 |  |  |  |  |
| *I am unable to walk about* |  | 0 | 0.0 |  |  |  | 0 | 0.0 |  |  |  |  |
| EQ-5D-5L Self-Care Subscale |  |  |  |  |  |  |  |  |  |  |  |  |
| Immediate Arm (Baseline, Week 4) | 40 |  |  |  |  | 28 |  |  |  |  | 0.30 | (0.03, 3.04) |
| *I have no problems washing or dressing myself* |  | 36 | 90.0 |  |  |  | 27 | 96.4 |  |  |  |  |
| *I have slight problems washing or dressing myself* |  | 4 | 10.0 |  |  |  | 1 | 3.6 |  |  |  |  |
| *I have moderate problems washing or dressing myself* |  | 0 | 0.0 |  |  |  | 0 | 0.0 |  |  |  |  |
| *I have severe problems washing or dressing myself* |  | 0 | 0.0 |  |  |  | 0 | 0.0 |  |  |  |  |
| *I am unable to wash or dress myself* |  | 0 | 0.0 |  |  |  | 0 | 0.0 |  |  |  |  |
| Immediate Arm (Baseline, Week 8) | 40 |  |  |  |  | 31 |  |  |  |  | 0.30 | (0.03, 3.04) |
| *I have no problems washing or dressing myself* |  | 36 | 90.0 |  |  |  | 30 | 96.8 |  |  |  |  |
| *I have slight problems washing or dressing myself* |  | 4 | 10.0 |  |  |  | 1 | 3.2 |  |  |  |  |
| *I have moderate problems washing or dressing myself* |  | 0 | 0.0 |  |  |  | 0 | 0.0 |  |  |  |  |
| *I have severe problems washing or dressing myself* |  | 0 | 0.0 |  |  |  | 0 | 0.0 |  |  |  |  |
| *I am unable to wash or dress myself* |  | 0 | 0.0 |  |  |  | 0 | 0.0 |  |  |  |  |
| Delayed Arm (Week 4, Week 8) | 38 |  |  |  |  | 31 |  |  |  |  | 0.23 | (0.03, 1.67) |
| *I have no problems washing or dressing myself* |  | 33 | 86.8 |  |  |  | 30 | 96.8 |  |  |  |  |
| *I have slight problems washing or dressing myself* |  | 5 | 13.2 |  |  |  | 0 | 0.0 |  |  |  |  |
| *I have moderate problems washing or dressing myself* |  | 0 | 0.0 |  |  |  | 1 | 3.2 |  |  |  |  |
| *I have severe problems washing or dressing myself* |  | 0 | 0.0 |  |  |  | 0 | 0.0 |  |  |  |  |
| *I am unable to wash or dress myself* |  | 0 | 0.0 |  |  |  | 0 | 0.0 |  |  |  |  |
| EQ-5D-5L Usual Activities Subscale |  |  |  |  |  |  |  |  |  |  |  |  |
| Immediate Arm (Baseline, Week 4) | 40 |  |  |  |  | 28 |  |  |  |  | 0.07** | (0.01, 0.38) |
| *I have no problems doing my usual activities* |  | 22 | 55.0 |  |  |  | 24 | 85.7 |  |  |  |  |
| *I have slight problems doing my usual activities* |  | 15 | 37.5 |  |  |  | 4 | 14.3 |  |  |  |  |
| *I have moderate problems doing my usual activities* |  | 2 | 5.0 |  |  |  | 0 | 0.0 |  |  |  |  |
| *I have severe problems doing my usual activities* |  | 1 | 2.5 |  |  |  | 0 | 0.0 |  |  |  |  |
| *I am unable to do my usual activities* |  | 0 | 0.0 |  |  |  | 0 | 0.0 |  |  |  |  |
| Immediate Arm (Baseline, Week 8) | 40 |  |  |  |  | 31 |  |  |  |  | 0.17* | (0.04, 0.66) |
| *I have no problems doing my usual activities* |  | 22 | 55.0 |  |  |  | 24 | 77.4 |  |  |  |  |
| *I have slight problems doing my usual activities* |  | 15 | 37.5 |  |  |  | 7 | 22.6 |  |  |  |  |
| *I have moderate problems doing my usual activities* |  | 2 | 5.0 |  |  |  | 0 | 0.0 |  |  |  |  |
| *I have severe problems doing my usual activities* |  | 1 | 2.5 |  |  |  | 0 | 0.0 |  |  |  |  |
| *I am unable to do my usual activities* |  | 0 | 0.0 |  |  |  | 0 | 0.0 |  |  |  |  |
| Delayed Arm (Week 4, Week 8) | 38 |  |  |  |  | 31 |  |  |  |  | 0.20* | (0.05, 0.85) |
| *I have no problems doing my usual activities* |  | 23 | 60.5 |  |  |  | 26 | 83.9 |  |  |  |  |
| *I have slight problems doing my usual activities* |  | 10 | 26.3 |  |  |  | 3 | 9.7 |  |  |  |  |
| *I have moderate problems doing my usual activities* |  | 4 | 10.5 |  |  |  | 1 | 3.2 |  |  |  |  |
| *I have severe problems doing my usual activities* |  | 1 | 2.6 |  |  |  | 0 | 0.0 |  |  |  |  |
| *I am unable to do my usual activities* |  | 0 | 0.0 |  |  |  | 1 | 3.2 |  |  |  |  |
| EQ-5D-5L Pain / Discomfort Subscale |  |  |  |  |  |  |  |  |  |  |  |  |
| Immediate Arm (Baseline, Week 4) | 40 |  |  |  |  | 28 |  |  |  |  | 0.73 | (0.20, 2.65) |
| *I have no pain or discomfort* |  | 15 | 37.5 |  |  |  | 15 | 53.6 |  |  |  |  |
| *I have slight pain or discomfort* |  | 19 | 47.5 |  |  |  | 9 | 32.1 |  |  |  |  |
| *I have moderate pain or discomfort* |  | 5 | 12.5 |  |  |  | 4 | 14.3 |  |  |  |  |
| *I have severe pain or discomfort* |  | 1 | 2.5 |  |  |  | 0 | 0.0 |  |  |  |  |
| *I have extreme pain or discomfort* |  | 0 | 0.0 |  |  |  | 0 | 0.0 |  |  |  |  |
| Immediate Arm (Baseline, Week 8) | 40 |  |  |  |  | 31 |  |  |  |  | 0.80 | (0.23, 2.80) |
| *I have no pain or discomfort* |  | 15 | 37.5 |  |  |  | 15 | 48.4 |  |  |  |  |
| *I have slight pain or discomfort* |  | 19 | 47.5 |  |  |  | 10 | 32.3 |  |  |  |  |
| *I have moderate pain or discomfort* |  | 5 | 12.5 |  |  |  | 6 | 19.4 |  |  |  |  |
| *I have severe pain or discomfort* |  | 1 | 2.5 |  |  |  | 0 | 0.0 |  |  |  |  |
| *I have extreme pain or discomfort* |  | 0 | 0.0 |  |  |  | 0 | 0.0 |  |  |  |  |
| Delayed Arm (Week 4, Week 8) | 38 |  |  |  |  | 31 |  |  |  |  | 0.21* | (0.05, 0.78) |
| *I have no pain or discomfort* |  | 14 | 36.8 |  |  |  | 18 | 58.1 |  |  |  |  |
| *I have slight pain or discomfort* |  | 19 | 50.0 |  |  |  | 10 | 32.3 |  |  |  |  |
| *I have moderate pain or discomfort* |  | 4 | 10.5 |  |  |  | 2 | 6.5 |  |  |  |  |
| *I have severe pain or discomfort* |  | 1 | 2.6 |  |  |  | 1 | 3.2 |  |  |  |  |
| *I have extreme pain or discomfort* |  | 0 | 0.0 |  |  |  | 0 | 0.0 |  |  |  |  |
| EQ-5D-5L Anxiety / Depression Subscale |  |  |  |  |  |  |  |  |  |  |  |  |
| Immediate Arm (Baseline, Week 4) | 40 |  |  |  |  | 28 |  |  |  |  | 0.08*** | (0.03, 0.23) |
| *I am not anxious or depressed* |  | 4 | 10.0 |  |  |  | 12 | 42.9 |  |  |  |  |
| *I am slightly anxious or depressed* |  | 11 | 27.5 |  |  |  | 11 | 39.3 |  |  |  |  |
| *I am moderately anxious or depressed* |  | 18 | 45.0 |  |  |  | 3 | 10.7 |  |  |  |  |
| *I am severely anxious or depressed* |  | 5 | 12.5 |  |  |  | 2 | 7.1 |  |  |  |  |
| *I am extremely anxious or depressed* |  | 2 | 5.0 |  |  |  | 0 | 0.0 |  |  |  |  |
| Immediate Arm (Baseline, Week 8) | 40 |  |  |  |  | 31 |  |  |  |  | 0.06*** | (0.02, 0.18) |
| *I am not anxious or depressed* |  | 4 | 10.0 |  |  |  | 14 | 45.2 |  |  |  |  |
| *I am slightly anxious or depressed* |  | 11 | 27.5 |  |  |  | 12 | 38.7 |  |  |  |  |
| *I am moderately anxious or depressed* |  | 18 | 45.0 |  |  |  | 4 | 12.9 |  |  |  |  |
| *I am severely anxious or depressed* |  | 5 | 12.5 |  |  |  | 1 | 3.2 |  |  |  |  |
| *I am extremely anxious or depressed* |  | 2 | 5.0 |  |  |  | 0 | 0.0 |  |  |  |  |
| Delayed Arm (Week 4, Week 8) | 38 |  |  |  |  | 31 |  |  |  |  | 0.13*** | (0.05, 0.37) |
| *I am not anxious or depressed* |  | 5 | 13.2 |  |  |  | 15 | 48.4 |  |  |  |  |
| *I am slightly anxious or depressed* |  | 17 | 44.7 |  |  |  | 11 | 35.5 |  |  |  |  |
| *I am moderately anxious or depressed* |  | 14 | 36.8 |  |  |  | 4 | 12.9 |  |  |  |  |
| *I am severely anxious or depressed* |  | 1 | 2.6 |  |  |  | 1 | 3.2 |  |  |  |  |
| *I am extremely anxious or depressed* |  | 1 | 2.6 |  |  |  | 0 | 0.0 |  |  |  |  |
|  | n | Mean | SD | Median | IQR | n | Mean | SD | Median | IQR | Mean Difference | 95% CI |
| EQ-5D-5L - Total Score |  |  |  |  |  |  |  |  |  |  |  |  |
| Immediate Arm (Baseline, Week 4) | 40 | 66.03 | 19.40 | 70.50 | 50.0 - 80.0 | 28 | 78.68 | 13.52 | 81.50 | 73.0 - 89.5 | 9.54** | (4.05, 15.04) |
| Immediate Arm (Baseline, Week 8) | 40 | 66.03 | 19.40 | 70.50 | 50.0 - 80.0 | 31 | 76.29 | 14.83 | 76.00 | 65.0 - 90.0 | 8.41** | (2.91, 13.91) |
| Delayed Arm (Week 4, Week 8) | 38 | 65.13 | 21.51 | 70.50 | 50.0 - 81.0 | 31 | 78.23 | 18.17 | 82.00 | 71.0 - 90.0 | 11.76*** | (5.84, 17.67) |
| PSYCHLOPS (impact of intrusive memories) |  |  |  |  |  |  |  |  |  |  |  |  |
| Immediate Arm (Baseline, Week 4) | 40 | 14.20 | 3.30 | 14.00 | 12.5 - 16.5 | 30 | 6.97 | 4.78 | 7.00 | 3.0 - 11.0 | -7.49*** | (-8.93, -6.05) |
| Immediate Arm (Baseline, Week 8) | 40 | 14.20 | 3.30 | 14.00 | 12.5 - 16.5 | 31 | 5.65 | 4.29 | 5.00 | 2.0 - 9.0 | -8.28*** | (-9.73, -6.83) |
| Delayed Arm (Week 4, Week 8) | 38 | 12.13 | 3.57 | 12.00 | 9.0 - 15.0 | 31 | 6.03 | 4.13 | 6.00 | 2.0 - 9.0 | -6.17*** | (-7.33, -5.02) |
|  |  |  |  |  |  |  |  |  |  |  |  |  |
| **Weekly Work Pattern** |  |  |  |  |  |  |  |  |  |  |  |  |
|  | n | Mean | SD | Median | IQR | n | Mean | SD | Median | IQR | Incident Rate Ratio | 95% CI |
| On how many days did you work this week? |  |  |  |  |  |  |  |  |  |  |  |  |
| Immediate Arm (Baseline, Week 4) | 42 | 3.21 | 1.80 | 4.00 | 2.0 - 5.0 | 35 | 2.66 | 2.13 | 3.00 | 0.0 - 4.0 | 0.83 | (0.64, 0.18) |
| Immediate Arm (Baseline, Week 8) | 42 | 3.21 | 1.80 | 4.00 | 2.0 - 5.0 | 31 | 3.48 | 1.84 | 4.00 | 3.0 - 5.0 | 1.11 | (0.85, 1.44) |
| Delayed Arm (Week 4, Week 8) | 38 | 3.26 | 1.78 | 4.00 | 2.0 - 5.0 | 30 | 2.83 | 1.58 | 3.00 | 2.0 - 4.0 | 0.88 | (0.66, 1.18) |
| How many times did you do a night shift this week? |  |  |  |  |  |  |  |  |  |  |  |  |
| Immediate Arm (Baseline, Week 4) | 42 | 1.00 | 1.36 | 0.00 | 0.0 - 3.0 | 35 | 0.83 | 1.25 | 0.00 | 0.0 - 2.0 | 0.80 | (0.50, 1.28) |
| Immediate Arm (Baseline, Week 8) | 42 | 1.00 | 1.36 | 0.00 | 0.0 - 3.0 | 31 | 0.77 | 1.18 | 0.00 | 0.0 - 2.0 | 0.80 | (0.48, 1.33) |
| Delayed Arm (Week 4, Week 8) | 38 | 0.55 | 1.01 | 0.00 | 0.0 - 1.0 | 30 | 0.57 | 0.97 | 0.00 | 0.0 - 1.0 | 1.06 | (0.53, 2.11) |
| ^1^Responses were made on an 11-point scale where 0 = not at all and 10 = extremely or very much.  *Notes.* n=total number observed; SD = Standard Deviation; IQR = Interquartile Range; ITT = Intention to Treat; CI = Confidence Interval; Freq. = Frequency of ordinal response items.  PCL-5 = PTSD Checklist for DSM-5 (4-item version); SCI-08 = Sleep Condition Indicator (8-item version); GAD-2 = Generalised Anxiety Disorder Assessment (2-item version); PHQ-2 = Patient Health Questionnaire (2-item version); SWEBO = Scale of Work Engagement and Burnout; WHODAS = World Health Organization Disability Assessment Schedule 2.0; EQ-5D-5L = European Quality of Life Five Dimension Five Level Scale; PSYCHLOPS = Psychological Outcome Profiles Questionnaire.  *ITT treatment effect estimates (treatment effects for within-group comparisons of each outcome variable):* Incidence Rate Ratio was utilised to analyse count data, where value < 1 indicates a lower rate post-intervention compared to pre-intervention.  Odds Ratio was utilised to analyse ordinal data, where value < 1 indicates lower odds post-intervention compared to pre-intervention.  Mean difference (pre- vs post-intervention) of change of score from baseline for ‘(Week 4, Week 8)’, and mean change of score for ‘(Baseline, Week 4)’ and for ‘(Baseline, Week 8)’, where value < 0 indicates a lower mean difference post-intervention compared to pre-intervention.  *Measure Scores:*  Measures of ‘Clinical Symptoms’: for Insomnia (SCI-08), a higher score indicates a greater quality of sleep (i.e., lower insomnia symptomology), whereas for PTSD symptoms (PCL-5), Anxiety Symptoms (GAD-2), and Depression Symptoms (PHQ-2) a higher score indicates worse symptom severity.  Measures of ‘Work Functioning’: for work burnout (SWEBO), work engagement (SWEBO), Intention to leave job, and Sickness Absence, a higher score indicates greater levels of work burnout, work engagement, intention to leave job, and number of sick days respectively.  Measures of ‘Wellbeing’: for impact on health and disability (WHODAS), and impact of self-identified problems (PSYCHLOPS), a higher score indicates greater difficulties in relation to the impact. For quality-of-life score (EQ-5D-5L – Total Score), a higher score indicates a higher quality of life.  ****p* < .001; ***p* < .01; **p* < .05 | | | | | | | | | | | | |

**Supplementary Table 4.**

*Descriptive Statistics for Changes to Health and Work.*

|  | Delayed Arm | | | Immediate Arm | | | All Participants (Combined) | | |
| --- | --- | --- | --- | --- | --- | --- | --- | --- | --- |
|  | n | Frequency 'yes' | % | n | Frequency 'yes' | % | n | Frequency 'yes' | % |
| Have you experienced any additional stressful life events? | | | | | | | | | |
| Week 4 | 38 | 18 | 47.4 | 28 | 13 | 46.4 | 66 | 31 | 47.0 |
| Week 8 | 31 | 11 | 35.5 | 31 | 11 | 35.5 | 62 | 22 | 35.5 |
| Have you received new treatments? | | | | | | | | | |
| Week 4 | 38 | 4 | 10.5 | 28 | 4 | 14.3 | 66 | 8 | 12.1 |
| Week 8 | 31 | 4 | 12.9 | 31 | 5 | 16.1 | 62 | 9 | 14.5 |
| Have you had any untoward medical occurrences or other problems? | | | | | | | | | |
| Week 4 | 38 | 4 | 10.5 | 28 | 1 | 3.6 | 66 | 5 | 7.6 |
| Week 8 | 31 | 2 | 6.5 | 31 | 4 | 12.9 | 62 | 6 | 9.7 |
| Do you work in the same job? | | | | | | | | | |
| Week 4 | 38 | 35 | 92.1 | 28 | 26 | 92.9 | 66 | 61 | 92.4 |
| Week 8 | 31 | 30 | 96.8 | 31 | 28 | 90.3 | 62 | 58 | 93.5 |
| Do you work the same number of hours per week? | | | | | | | | | |
| Week 4 | 38 | 35 | 92.1 | 28 | 25 | 89.3 | 66 | 60 | 90.9 |
| Week 8 | 31 | 30 | 96.8 | 31 | 28 | 90.3 | 61 | 58 | 93.5 |
| *Notes.* All items consisted of dichotomous (‘yes’/’no’) responses. This table presents the frequency and proportion of ‘yes’ responses to items assessing changes to participants’ health and work situations post-baseline at weeks 4 and 8. | | | | | | | | | |

**Supplementary Table 5.**

*Descriptive Statistics for Feedback Questionnaire.*

| Questionnaire Item | Delayed Arm | | | | | Immediate Arm | | | | | All Participants (Combined) | | | | |  |
| --- | --- | --- | --- | --- | --- | --- | --- | --- | --- | --- | --- | --- | --- | --- | --- | --- |
|  | n | Mean | SD | Median | IQR | n | Mean | SD | Median | IQR | n | Mean | SD | Median | IQR |  |
| How easy did you find it to use the intervention?  (0 = not at all easy – 10 = very easy) | 28 | 8.82 | 1.61 | 9.5 | 8.0 - 10.0 | 28 | 8.36 | 2.21 | 9.0 | 8.0 - 10.0 | 56 | 8.59 | 1.93 | 9.0 | 8.0 - 10.0 |  |
| How helpful did you find the intervention?  (0 = not at all helpful – 10 = very helpful) | 28 | 8.07 | 1.96 | 8.0 | 7.0 - 10.0 | 28 | 8.39 | 2.66 | 9.0 | 8.0 - 10.0 | 56 | 8.23 | 2.32 | 9.0 | 7.5 - 10.0 |  |
| How burdensome did you find the intervention?  (0 = very burdensome – 10 = not at all burdensome) | 28 | 6.36 | 2.63 | 7.0 | 5.0 - 8.0 | 28 | 6.61 | 2.82 | 7.0 | 5.5 - 8.5 | 56 | 6.48 | 2.70 | 7.0 | 5.0 - 8.0 |  |
| How distressing did you find the intervention?  (0 = very distressing – 10 = not at all distressing) | 28 | 7.00 | 2.74 | 7.5 | 5.0 - 9.5 | 28 | 7.14 | 2.58 | 7.5 | 6.0 - 9.5 | 56 | 7.07 | 2.63 | 7.5 | 5.5 - 9.5 |  |
| Overall, how acceptable did you find the intervention?  (0 = not at all acceptable – 10 = very acceptable) | 28 | 8.57 | 1.55 | 9.0 | 8.0 - 10.0 | 28 | 8.43 | 2.18 | 9.0 | 8.0 - 10.0 | 56 | 8.50 | 1.88 | 9.0 | 8.0 - 10.0 |  |
| If you were having intrusive memories in the future, how willing would you be to use the intervention if it was offered to you as something that would help?  (0 = not at all willing – 10 = very willing) | 28 | 8.71 | 1.92 | 10.0 | 7.5 - 10.0 | 28 | 8.86 | 2.32 | 10.0 | 9.0 - 10.0 | 56 | 8.79 | 2.11 | 10.0 | 8.0 - 10.0 |  |
| If a colleague or friend was having intrusive memories, how confident would you be in recommending the intervention to them?  (0 = not at all confident – 10 = very confident) | 28 | 8.14 | 2.22 | 9.0 | 6.5 - 10.0 | 28 | 8.71 | 2.23 | 10.0 | 8.0 - 10.0 | 56 | 8.43 | 2.22 | 10.0 | 7.5 - 10.0 |  |
| How much do you feel that this intervention could be used within NHS Trusts/healthcare organisations to support staff who have experienced work-related traumatic events?  (0 = not at all – 10 = very much) | 28 | 8.11 | 2.31 | 9.0 | 7.5 - 10.0 | 28 | 8.64 | 2.16 | 9.0 | 8.0 - 10.0 | 56 | 8.38 | 2.24 | 9.0 | 8.0 - 10.0 |  |
| Total Score | 28 | 63.79 | 11.54 | 66.0 | 57.0 - 72.0 | 28 | 65.14 | 13.58 | 70.0 | 63.0 - 73.5 | 56 | 64.46 | 12.51 | 67.0 | 60.0 - 72.5 |  |
| *Note.* Feedback questionnaire completed post-intervention (i.e., Week 4 for immediate arm, and Week 8 for delayed arm). SD = Standard Deviation; IQR = Interquartile Range. | | | | | | | | | | | | | | | | |

**Supplementary Table 6.**

*Between-group Analysis of Weekly Work Pattern*

|  | Delayed Arm | | | | | Immediate Arm | | | | | ITT Treatment Effect Estimate | |
| --- | --- | --- | --- | --- | --- | --- | --- | --- | --- | --- | --- | --- |
|  | n | Mean | SD | Median | IQR | n | Mean | SD | Median | IQR | IRR | 95% CI |
| On how many days did you work this week? |  |  |  |  |  |  |  |  |  |  |  |  |
| Baseline | 43 | 3.14 | 1.81 | 3.0 | 2.0 - 5.0 | 42 | 3.21 | 1.80 | 4.0 | 2.0 - 5.0 |  |  |
| Week 4 | 38 | 3.26 | 1.78 | 4.0 | 2.0 - 5.0 | 35 | 2.66 | 2.13 | 3.0 | 0.0 - 4.0 | 0.80 | (0.60, 1.07) |
| Week 8 | 30 | 2.83 | 1.58 | 3.0 | 2.0 - 4.0 | 31 | 3.48 | 1.84 | 4.0 | 3.0 - 5.0 |  |  |
| How many times did you do a night shift this week? |  |  |  |  |  |  |  |  |  |  |  |  |
| Baseline | 43 | 0.65 | 1.07 | 0 | 0.0 - 1.0 | 42 | 1.0 | 1.36 | 0.0 | 0.0 - 3.0 |  |  |
| Week 4 | 38 | 0.55 | 1.01 | 0 | 0.0 - 1.0 | 35 | 0.83 | 1.25 | 0.0 | 0.0 - 2.0 | 1.09 | (0.57, 2.09) |
| Week 8 | 30 | 0.57 | 0.97 | 0 | 0.0 - 1.0 | 31 | 0.77 | 1.18 | 0.0 | 0.0 - 2.0 |  |  |
| *Note.* Between-groups comparisons at week 4 did not reach statistical significance (for both, *p* > .05). ITT = Intention to Treat; SD = Standard Deviation; IQR = Interquartile Range; IRR = Incidence Rate Ratio; CI = Confidence Interval. | | | | | | | | | | | | |

**Supplementary Table 7.**

*Support from Managers, Family and Friends at Baseline.*

|  | Delayed Arm | | |  | Immediate Arm | | |  | All Participants (Combined) | | |
| --- | --- | --- | --- | --- | --- | --- | --- | --- | --- | --- | --- |
|  | n | Freq. | % |  | n | Freq. | % |  | n | Freq. | % |
| How well supported have you been by your supervisors/managers? | 42 |  |  |  | 41 |  |  |  | 83 |  |  |
| *Not at all* |  | 9 | 21.4 |  |  | 6 | 14.6 |  |  | 15 | 18.1 |
| *A little bit* |  | 14 | 33.3 |  |  | 15 | 36.6 |  |  | 29 | 34.9 |
| *Moderately* |  | 10 | 23.8 |  |  | 9 | 22.0 |  |  | 19 | 22.9 |
| *Quite a bit* |  | 7 | 16.7 |  |  | 7 | 17.1 |  |  | 14 | 16.9 |
| *Extremely* |  | 2 | 4.8 |  |  | 4 | 9.8 |  |  | 6 | 7.2 |
| How well supported have you been by your family & friends? | 42 |  |  |  | 41 |  |  |  | 83 |  |  |
| *Not at all* |  | 1 | 2.4 |  |  | 1 | 2.4 |  |  | 2 | 2.4 |
| *A little bit* |  | 2 | 4.8 |  |  | 10 | 24.4 |  |  | 12 | 14.5 |
| *Moderately* |  | 11 | 26.2 |  |  | 10 | 24.4 |  |  | 21 | 25.3 |
| *Quite a bit* |  | 15 | 35.7 |  |  | 9 | 22.0 |  |  | 24 | 28.9 |
| *Extremely* |  | 13 | 31.0 |  |  | 11 | 26.8 |  |  | 24 | 28.9 |
| *Note*. n = total number of observed responses; Freq. = Frequency of categorial response items. | | | | | | | | | | | |

**Supplementary Table 8.**

*Summary of Adverse Events.*

| Adverse Event Reported | | Immediate Arm | | Delayed Arm | |
| --- | --- | --- | --- | --- | --- |
|  |  | Number | | Number | |
|  | |  | |  | |
| Spinal procedure | | 0 | | 1 | |
| Recent leg surgery | | 1 | | 0 | |
| Deranged bloods | | 0 | | 1 | |
| Joint pain | | 2 | | 0 | |
| Headache | | 0 | | 1 | |
| Worsening of a gynae issue | | 1 | | 0 | |
| Endometriosis | | 1 | | 0 | |
| Breast lump | | 0 | | 1 | |
| Menopause symptoms | | 0 | | 1 | |
| Cardiac issues | | 0 | | 1 | |
| Viral infection (Not Otherwise Specified) | | 1 | | 1 | |
| Acute gastro-enteritis | | 1 | | 0 | |
| Chest infection and tonsilitis | | 1 | | 0 | |
| Contracted COVID | | 2 | | 4 | |
| Respiratory virus (Not Otherwise Specified) | | 0 | | 1 | |
| Chest infection | | 0 | | 1 | |
| Worsening mental health | | 0 | | 1 | |
| Suicidal ideation | | 1 | | 0 | |
| *Responses to Changes to Health and Work: “Have you received any new treatments?”* | | | | | |
| Propanol | | 1 | | 1 | |
| Fluoxetine | | 0 | | 1 | |
| Anxiety medication (Not Otherwise Specified) | | 0 | | 1 | |
| Amitriptyline | | 0 | | 1 | |
| Accessed counselling | | 1 | | 1 | |
| Total Number of Adverse Events | | 13 | | 19 | |
| **Serious Adverse Event** | | | | | |
|  | Immediate Arm | | Delayed Arm | | Outcome |
| Symptom | Number | | Number | |  |
| Admitted to hospital due to a ‘chest infection with reduced foetal movement’ | 1 | | 0 | | Participation in the study was paused |
| *Note.* All adverse events and serious adverse events were unrelated to the study intervention or procedures. No participants were withdrawn due to adverse events. In the immediate arm 13 adverse events in 11 participants were recorded, and in the delayed arm 19 adverse events in 14 participants were recorded. In the immediate arm, 1 serious adverse event was reported – participation in the study was paused for this participant. Adverse events were reported by participants either when completing secondary outcome questionnaires at baseline, week 4 and week 8, or were reported during contact with researchers. The percentage of participants in either arm reporting an adverse event was 25.58%. | | | | | |
